# The Long-Term Efficacy of Cephalosporin in Elderly Hip Fracture Patients: A Comprehensive Analysis

**DOI:** 10.3390/jcm14176086

**Published:** 2025-08-28

**Authors:** Huiqing Pan, Xiao Wang, Qingjian Ou, Juan Wang, Zisheng Ai

**Affiliations:** School of Medicine, Tongji University, Shanghai 200065, China

**Keywords:** antibiotic prophylaxis, cephalosporin, monotherapy, combination therapy, hip fracture

## Abstract

**Objectives**: This study aims to evaluate the association between antibiotic prophylaxis (particularly cephalosporins) and clinical outcomes in elderly hip fracture patients. **Methods:** We analyzed 4044 elderly hip fracture patients (2008–2022) from the Medical Information Mart for Intensive Care IV (MIMIC-IV) database using inverse probability treatment weighting (IPTW). Cox proportional hazards models assessed mortality risk, while logistic regression evaluated infection and Intensive Care Unit (ICU) admission risks. Dose–response and subgroup analyses were performed for significant findings. **Results:** In total, 166 patients received no antibiotics, 2589 received Cephalosporin monotherapy, 403 received non-cephalosporin therapy, and 886 received Cephalosporin combination therapy. After IPTW adjustment, monotherapy showed significantly lower mortality risk versus combination therapy at all timepoints (hazard ratio (HR) for 28-day mortality: 0.46, 95% confidence interval (95% CI): 0.28–0.75; HR for 90-day mortality: 0.60, 95% CI: 0.44–0.82; HR for 180-day mortality: 0.67, 95% CI: 0.51–0.87; HR for 1-year mortality: 0.71, 95% CI: 0.57–0.89). The SII cut-off values were 1310.1 for 28-day mortality, 2077.5 for both 90-day and 180-day mortality, 1742.2 for 1-year mortality, 2199.7 for ICU admission, and 1930.7 for infection. Subgroup analyses showed that males and internal fixation patients derived more benefits after cephalosporin monotherapy treatment at all time nodes. Patients with multiple injuries had a lower risk of 28-day mortality, while high-comorbidity patients (CCI ≥ 5) and those with osteoporosis exhibited particular advantages with cephalosporin monotherapy. **Conclusions:** Cephalosporin monotherapy appears non-inferior to combination therapy for elderly hip fracture patients, potentially reducing long-term mortality risk, especially in males, internal fixation cases, and patients with CCI ≥ 5 and osteoporosis.

## 1. Introduction

Hip fractures are common in older adults, with 20% to 30% of patients succumbing within the subsequent year [1,2]. Surgery, including internal fixation and hip replacement, continues to be the principal therapeutic modality [3]. Approximately 10% of patients develop surgical site infections (SSIs) after hip fracture repair, potentially resulting in increased mortality, Intensive Care Unit (ICU) admission, and additional costs [4,5]. Current guidelines recommend perioperative antibiotic prophylaxis, such as first- and second-generation cephalosporins (e.g., cefazolin), as a potential first-line option to prevent SSIs and reduce mortality [6]. Alternative medications may be utilized in particular circumstances, such as clindamycin or vancomycin for individuals with β-lactam allergies, or teicoplanin in environments with elevated prevalence of methicillin-resistant S. aureus (MRSA) or confirmed colonization [7]. Combination treatments, such as a cephalosporin plus vancomycin or gentamicin [6], have been investigated to broaden coverage against resistant Gram-positive or Gram-negative pathogens; nevertheless, the hazards of nephrotoxicity and resistance necessitate thorough evaluation. In summary, although various antibiotics are accessible for prophylaxis in hip surgery, cephalosporins are the preferred option for the majority of patients owing to their favorable antibacterial range, tolerability, cost-effectiveness, and robust evidence-based endorsement. Cephalosporins, a class of β-lactam antibiotics, inhibit bacterial cell wall synthesis and are effective against Staphylococcus aureus and coagulase-negative staphylococci, with additional coverage against select Gram-negative pathogens [8,9]. Perioperative use of cephalosporins was associated with a 5.01% reduction in deep SSI risk and a 24% lower risk of 1-year mortality [5].

The abuse and misuse of cephalosporins are prevalent in clinical practice. Broad-spectrum antibiotic use, including the combination of cephalosporins and other drugs (e.g., gentamicin), has risen significantly, reaching 72.56 Defined Daily Doses (DDDs) per 100 bed-days [10,11]. A study revealed that merely three out of five hip replacement surgeries adhered to correct antibiotic protocols [7]. The abuse and misuse of cephalosporins exacerbate the escalating issue of antibiotic resistance, potentially leading to refractory infections and higher mortality rates. A study projected that 8.22 million deaths could be linked to antimicrobial resistance by 2050 [12]. The effectiveness of various antibiotic regimens is still contested. A meta-analysis indicated no distinction between combination therapy and monotherapy [13]. Another found that a single preoperative dose of cephalosporin plus gentamicin reduced C. difficile-associated disease and mortality [14]. Consequently, maximizing the utilization of cephalosporins is essential.

Given the high incidence of antimicrobial resistance and the uncertainty in agent selection, it is crucial to evaluate the impacts of antibiotic prophylaxis, particularly cephalosporins, on survival consequences in various time nodes and to compare the effectiveness of different regimens in elderly hip fracture patients. Although some studies have suggested short-term benefits and similar efficacy of cephalosporins with teicoplanin, cephalosporins, and penicillin derivatives [15], the results were inconsistent and focused on short-term outcomes. Evidence on long-term consequences in elderly hip fracture patients who received cephalosporins remains limited, which might be important. A meta-analysis indicated that antibiotic treatment might enhance the long-term quality of survival for older patients with hip fractures; however, the sample size was insufficient to achieve statistical significance [16]. Furthermore, the application of combination therapy may occasionally be unnecessary. The efficacy of cephalosporin combination therapy requires detailed evaluation in a large sample size. Consequently, further investigation is required to examine the long-term mortality trends following antibiotic prophylaxis, to comprehensively compare the efficacy of cephalosporin monotherapy against combination therapy, and to determine the appropriate treatment regimes.

In this study, we hypothesized that cephalosporin monotherapy would demonstrate non-inferiority to combination therapy in the risks of infection, ICU admission, and mortality at successive timepoints—28 days, 90 days, 180 days, and 1 year—in elderly patients with hip fractures. We conduct a retrospective analysis of elderly hip fracture patients to evaluate the risks of survival outcomes after antibiotic use, with a focus on cephalosporins. The study aims to observe the long-term correlation between cephalosporin utilization and mortality from 2008 to 2022, to assess whether cephalosporin exposure mitigates mortality and the associated risks, and to conduct a comprehensive comparison of the efficacy of cephalosporin monotherapy versus combination therapy. Additionally, we analyze the dose–response relationships across regimens by using the DDDs. Finally, we conduct subgroup and sensitivity analyses. These findings will guide clinical measures to mitigate adverse outcomes following hip fracture surgery.

## 2. Materials and Methods

### 2.1. Study Population

This retrospective observational study utilized data from the Medical Information Mart for Intensive Care IV (MIMIC-IV) database, a publicly available critical care repository comprising over 60,000 ICU admissions at a U.S. tertiary academic medical center between 2009 and 2022 [17]. One author (Huiqing Pan; certification number: 12093395) completed the Collaborative Institutional Training Initiative (CITI) examination to access the database. The project was approved by the institutional review boards of Beth Israel Deaconess Medical Center and Massachusetts Institute of Technology. All participants in this study remained anonymous, so additional ethics committee approval was waived for this study. The article adhered to the Strengthening the Reporting of Observational Studies in Epidemiology (STROBE) statement [18].

**Figure 1:**
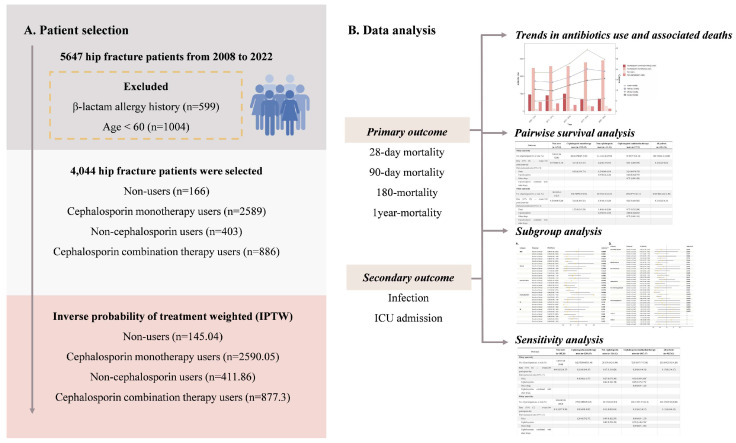
Flowchart of patient inclusion and process of data analysis. (**A**) Flowchart of patient inclusion. (**B**) Process of data analysis. * Statistically significant according to the *p*-value adjusted by FDR.

The inclusion criteria (Figure 1A) were elderly hip fracture patients who had received internal fixation or hip replacement and who had complete medical records. We identified hip fracture patients according to the *International Classification of Diseases 11th Revision* (ICD-11) (NC72) [19]. If a patient had multiple hospital admissions, only the data from the first admission were included in the analysis. Patients with a β-lactam allergy history were excluded. Patients aged < 60 years were also excluded.

### 2.2. Data Collection

PostgreSQL (ver. 11.0) was used to extract all the variables and outcomes in a Structured Query Language (SQL) format. We retrieved demographic and administrative data (age, gender, anchor year, race, admission type, BMI, and blood pressure), laboratory examinations (red blood cell, white blood cell, neutrophil count, lymphocyte count, platelet, hemoglobin, creatinine, BUN, chloride, bicarbonate, potassium, sodium, anion gap, and glucose), medical history (mechanical ventilation, renal replacement therapy, vasopressin use, immunosuppressant use, and types of fracture surgeries), comorbidity (Charlson comorbidity index (CCI), osteoporosis, and multiple injuries), and detailed antibiotic administration records, including total doses and specific drug categories. We calculated the Systemic Immune-Inflammation Index (SII) through the following formula: platelet count × neutrophil count/lymphocyte count [20]. We categorized antibiotics into four groups: non-users (group 1), cephalosporin monotherapy users (group 2), non-cephalosporin users (group 3), and cephalosporin combination therapy users (group 4). Cephalosporins were identified according to the Anatomical Therapeutic Chemical (ATC) classification system [21].

DDD is a statistical measure of drug consumption defined by the World Health Organization. The DDD is the assumed average daily maintenance dose for a drug’s primary indication in adults, which is used to standardize antibiotic consumption comparisons across regimens and settings. Following established methodology [22], we calculated the number of DDDs by using the following formula: (total amount of drug)/(amount of drug in a DDD) [21]. To evaluate dose–response relationships, we computed the cumulative defined daily dose (cDDD) for each patient by summing the dispensed DDD of all administered antibiotics during hospitalization.

### 2.3. Outcome Definition

The primary outcomes of interest were 28-day, 90-day, 180-day, and 1-year all-cause mortality, and all of the primary outcomes could be directly extracted from the MIMIC database. The secondary outcomes were infection during hospitalization and ICU admission.

### 2.4. Statistical Analysis

Patient characteristics were reported as mean ± standard deviation (SD), median (interquartile range), or number (percentage). Group comparisons were performed using the *χ^2^* test for categorical variables and the *t*-test or the Mann–Whitney *U* test for continuous variables. We used Multiple Imputation by Chained Equations for filling missing values. We used line charts and bar charts to visualize trends in antibiotic use and mortality rates from 2008 to 2022 after population standardization.

To enhance comparability among the four antibiotic exposure groups and avoid potential bias, we conducted inverse probability of treatment weighted (IPTW) analyses [23]. The weights were estimated using propensity score weighting via generalized linear models (GLMs) implemented through the WeightIt package (ver. 1.4.0), which serves as a unified interface for various balancing weight estimation methods. The GLM-based approach (‘ps’ method) was specifically employed, as it flexibly accommodates multinomial treatment variables. The model was adjusted for multiple potential confounders, including vital signs, comorbidity, and laboratory data. To improve weight efficiency, we applied stabilization to the estimated weights. Kaplan–Meier plots were used to capture the cumulative incidence of outcomes according to the time from hospital admission to the visit at which an event was first reported and subsequently confirmed. Cox proportional hazards models were used to estimate the hazard ratios (HRs) for associations between antibiotic use and mortality risks in both univariate and multivariate analyses. Logistic regression models were used to examine the associations of antibiotic use with ICU admission and infection risks. For significant pairwise comparisons, restricted cubic spline models with three knots and the non-linear test were used to assess the potential non-linear relationship [24].

For survival and binary outcomes, since we compared four groups, we implemented post hoc pairwise comparisons using the emmeans package, with estimated marginal means and associated 95% confidence intervals (95% CIs) [25]. The results of all analyses were expressed as HRs or odds ratios (ORs), all with accompanying 95% CIs [26]. Since multiple studies have shown that SII is independently associated with short-term and long-term mortality in elderly patients with hip fractures [27], we calculated ROC/Youden to identify the optimal cut-off value for SII. Subgroup analyses were performed according to the prescribed factors, including gender, surgery, Charlson comorbidity index (CCI), SII, immunosuppressant use, multiple injuries, and osteoporosis [28]. Interaction effects between subgroups were tested using Wald tests. To account for multiple comparisons, we used the false discovery rate (FDR) correction method (Benjamini–Hochberg procedure) to adjust the *p*-value [29].

All analyses were performed at a two-sided significance level of α = 0.05. A two-tailed *p*-value < 0.05 was considered statistically significant. All statistical analyses were performed using the R software (ver. 4.4.2; R Foundation for Statistical Computing, Vienna, Austria) and Python software (ver. 3.11).

## 3. Results

### 3.1. Population Characteristics

Data used for analyses are presented in Supplementary Table S1. The patient selection procedure is shown in Figure 1, with a total of 4044 elderly hip fracture patients meeting the study criteria. There were 166 patients (4.1%) who did not receive antibiotic treatment (group 1), 2589 (64.0%) who received cephalosporin monotherapy (group 2), 403 (10.0%) who received non-cephalosporin therapy (group 3), and 886 (21.9%) who received cephalosporin combination therapy (group 4). The average age of the participants was 79.87 years. In total, 57 patients (34.3%) in group 1, 802 patients (31.0%) in group 2, 167 patients (41.4%) in group 3, and 423 patients (47.7%) in group 4 died. Data on 28-day, 90-day, 180-day, and 1-year mortality, infection, and ICU admission are shown in Supplementary Table S2. Among the four groups, variables such as laboratory data, comorbidity, and blood pressure were imbalanced. Baseline characteristics were balanced by applying the IPTW method. In total, 145.04 patients in group 1, 2590.05 patients in group 2, 411.86 patients in group 3, and 877.30 patients in group 4 were included, resulting in a final study cohort of 4024.24 patients. The baseline characteristics of IPTW patients among the four groups were similar, which can be seen in Table 1.

### 3.2. Antibiotic Use and Mortality Trends from 2008 to 2022

**Figure 2:**
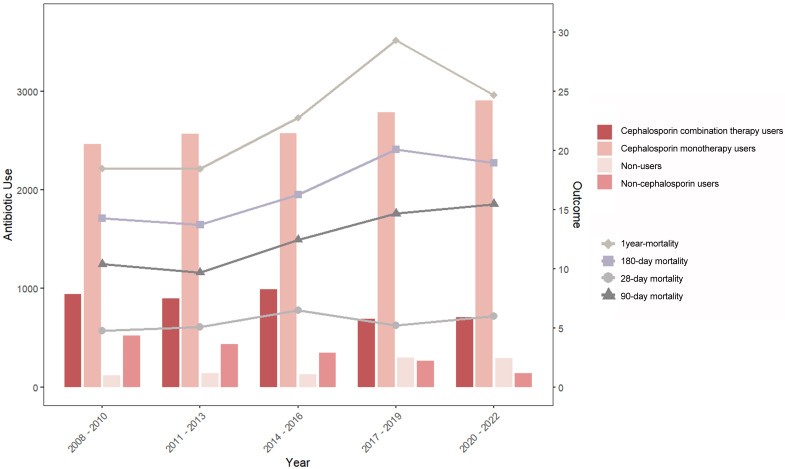
Antibiotic use and mortality trends from 2008 to 2022.

After adjusting for population structure, we analyzed temporal trends in treatment patterns and clinical outcomes from 2008 to 2022. Figure 2 showed that cephalosporin monotherapy utilization demonstrated a gradual increase over the study period. Mortality trends showed distinct temporal variations, with 28-day mortality exhibiting modest increases during 2014–2016 and 2020–2022, while 90-day mortality gradually increased between 2011 and 2022. The highest 180-day and 1-year mortality rates were observed during 2017–2019. An emerging trend was the growing proportion of patients opting for complete avoidance of perioperative antibiotic prophylaxis. Conversely, cephalosporin use became more prevalent among those receiving prophylaxis.

### 3.3. Association of Cephalosporins with Primary Outcomes

Table 2 shows the number of patients in each group who died in 28 days, 90 days, 180 days, and 1 year, and the corresponding rates, as well as the pairwise HRs among the four groups.

Among elderly hip fracture patients, the 28-day mortality rate was 4.90% in total, with the highest frequency in group 4 (8.10%) and the lowest frequency in group 1 (2.00%). The 90-day mortality rate was 11.3% in total, with the highest frequency in group 4 (15.4%) and the lowest frequency in group 2 (9.8%). The 180-day mortality rate was 15.4% in total, with the highest frequency in group 4 (19.3%), and the lowest frequency in group 2 (13.6%). The 1-year mortality rate was 20.7% in total, with the highest frequency in group 1 (25.2%) and the lowest frequency in group 2 (19.1%).

**Figure 3:**
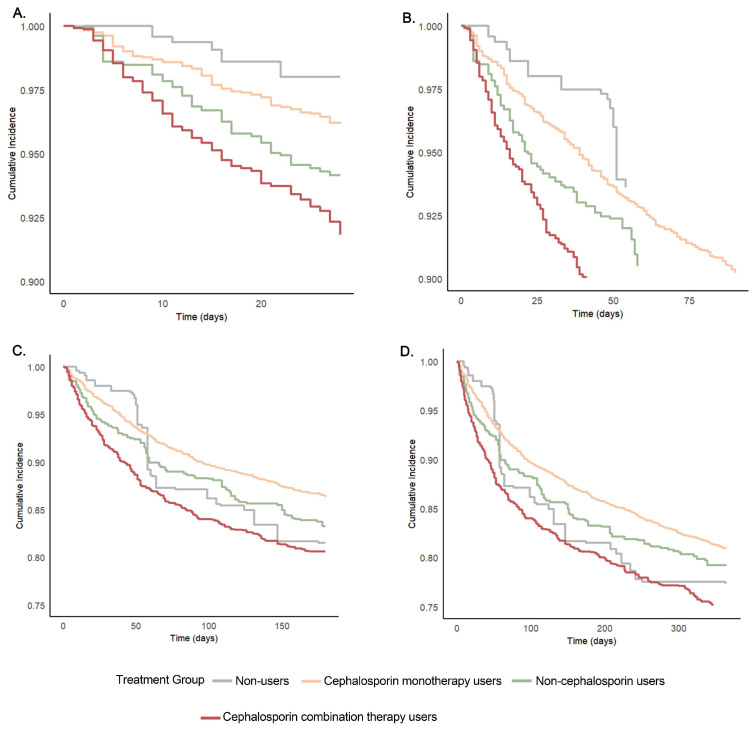
Kaplan–Meier estimates of the cumulative incidence of mortality: (**A**) 28-day mortality; (**B**) 90-day mortality; (**C**) 180-day mortality; (**D**) 1-year mortality.

The six pairwise comparisons among four groups showed reduced 28-day mortality risks for group 1 versus group 4 (HR, 0.24; 95% CI, 0.07–0.78), and group 2 versus group 4 (HR, 0.46; 95% CI, 0.28–0.75). The difference between group 1 and group 2 was not significant. The risk of 90-day mortality was 40% lower in group 2 than in group 4 (HR, 0.60; 95% CI, 0.44–0.82). Group 2 had a significantly lower risk of 180-day mortality than group 4 (HR, 0.67; 95% CI, 0.51–0.87). The risk of 1-year mortality was 29% higher in group 4 than in group 2 (HR, 0.71; 95% CI, 0.57–0.89). Figure 3 showed the between-group differences in the Kaplan–Meier estimates of the cumulative incidence of mortality, which were consistent with six pairwise comparisons.

**Figure 4:**
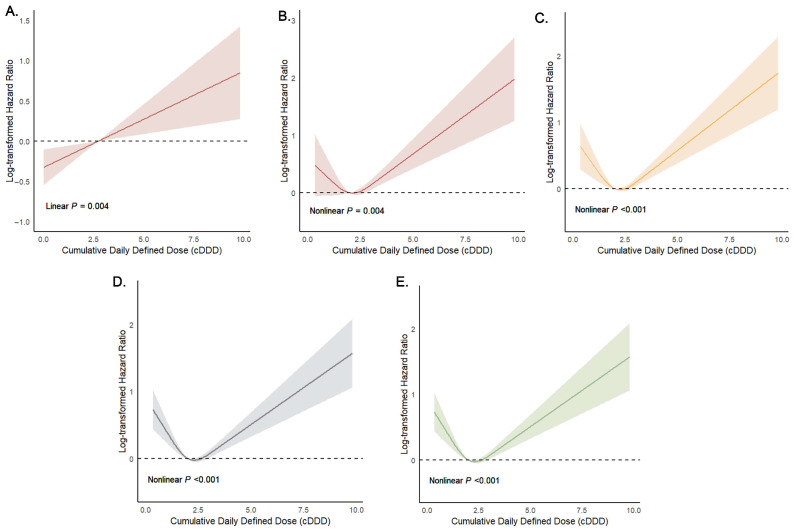
Dose–response association between the cDDD of antibiotics and primary outcomes: (**A**) 28-day mortality (comparison between group 1 and group 4); (**B**) 28-day mortality (comparison between group 2 and group 4); (**C**) 90-day mortality (comparison between group 2 and group 4); (**D**) 180-day mortality (comparison between group 2 and group 4); (**E**) 1-year mortality (comparison between group 2 and group 4).

We selected groups with pairwise significance to conduct dose–response associations between the cumulative dose and the primary outcomes. Figure 4 showed a non-linear relationship between the cumulative antibiotic dose and 28-day (group 2 and group 4), 90-day, 180-day, and 1-year mortality risk, with mortality initially decreasing then increasing at higher doses, while the 28-day mortality risk (group 1 and group 4) showed a linear dose–response relationship (non-linear test *p* > 0.05). These findings collectively demonstrated significant variations in clinical outcomes based on antibiotic treatment strategies in elderly hip fracture patients.

### 3.4. Association of Cephalosporins with Secondary Outcomes

Table 3 shows the number of patients in each group with infection and ICU admission, and the corresponding rates, as well as the pairwise ORs among the four groups. The infection rate was 5.6% in total, with the highest frequency in group 4 (6.0%) and the lowest in group 1 (0.5%). The overall ICU admission rate was 12.2%, with the highest frequency in group 3 (12.8%) and the lowest frequency in group 1 (6.5%). Assessments of the homogeneity of treatment-group differences showed that the pattern of risks across treatment groups did not differ materially.

### 3.5. Subgroup Analysis

**Figure 5:**
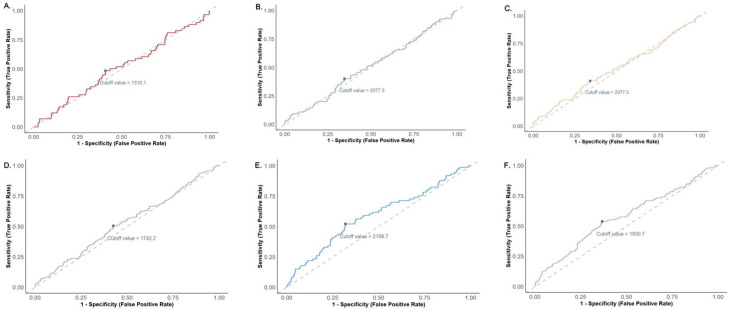
Cut-off values for SII related to both primary and secondary outcomes: (**A**) 28-day mortality; (**B**) 90-day mortality; (**C**) 180-day mortality; (**D**) 1-year mortality; (**E**) ICU admission; (**F**) Infection.

The optimal cut-off values for the SII were determined using ROC curve analysis with Youden’s index for six clinical outcomes (Figure 5). The SII cut-off value for 28-day mortality was 1310.1. Higher cut-off values were identified for longer-term mortality outcomes: 2077.5 for both 90-day and 180-day mortality rates and 1742.2 for 1-year mortality. Regarding secondary outcomes, SII demonstrated greater discriminatory thresholds for critical care needs (2199.7 for ICU admission) and infection (1930.7).

**Figure 6:**
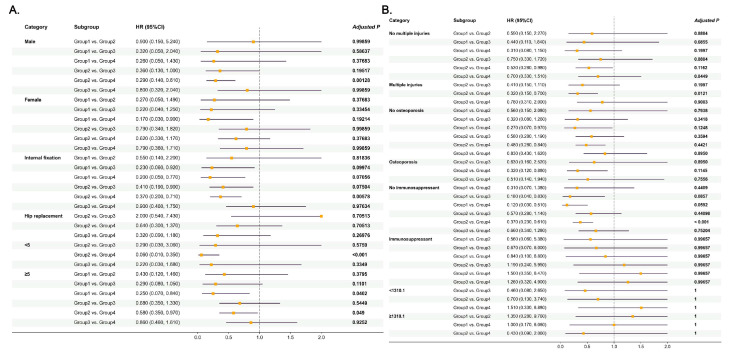
Associations between cephalosporin use and 28-day mortality stratified by subgroups, presented as adjusted HRs with 95% confidence intervals. (**A**) Subgroups based on: gender, surgery, and CCI. (**B**) Subgroups based on: multiple injuries, osteoporosis, immunosuppressant use, and SII.

Supplementary Tables S3–S5 present the descriptive primary and secondary outcomes stratified by subgroups. Cephalosporin monotherapy was associated with a lower risk of 28-day mortality in males (HR, 0.29; 95% CI, 0.14–0.61; adjusted *p*-value, 0.001) compared to female participants (HR, 0.62; 95% CI, 0.33–1.17; adjusted *p*-value, 0.377), as well as the reduction observed in those undergoing internal fixation (HR, 0.37; 95% CI, 0.20–0.71; adjusted *p*-value, 0.006) (Figure 6A). Similar trends were also observed for 90-day, 180-day, and 1-year mortality risks (Supplementary Figures S1A, S2A, and S3A). In patients with CCI ≥ 5, cephalosporin monotherapy users had lower risks of 180-day and 1-year mortality (180-day mortality risk: HR, 0.68, 95% CI, 0.52–0.90, adjusted *p*-value, 0.033; 1-year mortality risk, HR, 0.73, 95% CI, 0.57–0.92, adjusted *p*-value, 0.041) compared to combination therapy users (Supplementary Figures S2A and S3A). As shown in Figure 6B, patients with multiple injuries had a lower risk of 28-day mortality (HR, 0.32; 95% CI, 0.15–0.70; adjusted *p*-value, 0.012). Supplementary Figures S1B and S2B showed that the 90-day and 180-day mortality risks were lower in group 2 for patients with osteoporosis (90-day mortality risk: HR, 0.58, 95% CI, 0.31–0.78, adjusted *p*-value, 0.001; 180-day mortality risk: HR, 0.66, 95% CI, 0.38–0.80, adjusted *p*-value, <0.001). Those who did not receive immunosuppressants (HR, 0.37; 95% CI, 0.23–0.61; adjusted *p*-value, <0.001) had lower risks of 28-day mortality; similar trends were also observed for 90-day, 180-day, and 1-year mortality risks (Figure 6B, Supplementary Figures S1B, S2B, and S3B). Comparable associations were shown between cephalosporin use and mortality risks across different SII strata over time.

For ICU admission, the associations were similar in every stratum except the one stratified by CCI ≥ 5. Non-drug users had a lower risk than all other groups (group 1 vs. group 2: OR, 0.37, 95% CI, 0.18–0.78, adjusted *p*-value, 0.038; group 1 vs. group 3: HR, 0.32; 95% CI, 0.13–0.79, adjusted *p*-value, 0.038; group 1 vs. group 4: HR, 0.42; 95% CI, 0.20–0.86, adjusted *p*-value, 0.041) (Supplementary Figure S4). Finally, subgroup analysis of infection risk showed consistent associations across strata, with no interactions (*p* > 0.05) (Supplementary Figure S5 and Supplementary Table S6).

### 3.6. Sensitivity Analysis

After including patients aged 18 or above, the results remained consistent with the primary findings. As shown in Table 4, group 1 exhibited a lower 28-day mortality risk compared to group 4 (HR, 0.19; 95% CI, 0.05–0.68), as did group 2 versus 4 (HR, 0.45; 95% CI, 0.27–0.75). Similarly, group 2 demonstrated a 45% reduction in 90-day mortality risk relative to group 4 (HR, 0.55; 95% CI, 0.40–0.76), along with lower risks for 180-day mortality risk (HR, 0.60; 95% CI, 0.46–0.78) and 1-year mortality (HR, 0.63; 95% CI, 0.50–0.78). Table 5 showed no significant differences in ICU admission and infection risks across the four groups.

## 4. Discussion

Consistent with the previous guideline [6], our study suggested that cephalosporin monotherapy may not be inferior to combination therapy overall and could be suggested for routine orthopedic surgery. For 28-day mortality risk, the efficacies of non-prophylaxis and cephalosporin monotherapy were superior to cephalosporin combination therapy, with comparable efficacy between these two groups. For 90-day, 180-day, and 1-year mortality, cephalosporins showed survival benefits over other groups. Dose–response analysis revealed a U-shaped association between antibiotic dosage and mortality risks, except for a linear association between groups 1 and 4 regarding 28-day mortality. Notably, cephalosporin monotherapy provided long-term survival benefits in males, internal fixation patients, those with CCI ≥ 5, and osteoporosis patients, while patients with multiple injuries experienced only short-term advantages. Additionally, patients not on immunosuppressants derived greater survival benefits from monotherapy.

In elderly patients with hip fractures, the observed reduction in all-cause mortality with cephalosporin monotherapy could be attributed to both infectious and non-infectious causes. On the one hand, for infection-caused mortality, current evidence supports the infection-prevention efficacy of antibiotic prophylaxis in hip fracture repair and total joint replacement, particularly for procedures involving the implantation of foreign materials [30]. On the other hand, non-infectious causes of death are common and include cardiovascular events (e.g., postoperative myocardial infarction), multi-organ failure (e.g., progressive hepatic or renal dysfunction), thromboembolic complications (e.g., pulmonary embolism or deep vein thrombosis), and progression of underlying diseases such as chronic obstructive pulmonary disease. Several large-scale studies showed that cardiovascular diseases, accidental injuries, and cancer together account for 10% to 22% of mortality within 30 days to 1 year after fracture [31,32]. As detailed below, cephalosporin monotherapy might indirectly decrease the risk of non-infectious mortality by lowering hepatotoxicity or nephrotoxicity or by drug–drug interactions.

Monotherapy might have more pharmacokinetic advantages. The risk of pharmacokinetic interaction occurring between cephalosporins and other antibiotics depends on factors such as binding affinity for Sudlow site II of Human Serum Albumin (HSA), plasma drug concentrations, the drug formulation, and interpatient variability [33]. Cefazolin, for example, exhibits concentration-dependent protein binding (~85% at low concentrations vs. ~49% at high concentrations), which might further decline to 60–77% under pathological conditions, indicating altered protein-binding dynamics [34,35]. Since only the unbound fraction is pharmacologically active and able to penetrate tissues, competitive binding at HSA sites (e.g., with vancomycin) could transiently increase the free drug fraction but simultaneously accelerate elimination, thereby reducing sustained tissue exposure [36]. This phenomenon is particularly relevant when combining ceftriaxone with cyclosporine, where competitive HSA binding elevates unbound cyclosporine levels, potentially exacerbating nephrotoxicity [37].

For long-term survival outcomes, monotherapy might reduce antibiotic-related syndromes. Nephrotoxicity and hepatotoxicity were significantly lower after cephalosporin use than penicillin and nafcillin monotherapy and combination use of cephalosporin and gentamicin, while acquiring similar efficacy [38,39,40], showing the safety of cephalosporin prophylaxis. Moreover, a recent randomized trial further supported that adding vancomycin to cefazolin prophylaxis in MRSA-negative arthroplasty patients did not significantly reduce surgical site infections but increased adverse events (e.g., hypersensitivity) [41], indicating that routine vancomycin addition is unnecessary without MRSA risk factors. This lower toxicity profile and pharmacokinetic advantages might be particularly relevant in elderly hip fracture patients, in whom hepatic and renal reserve is often limited and who are already vulnerable to non-infectious complications such as cardiovascular events or multi-organ failure.

Our subgroup analysis revealed that male patients derived significant survival benefits from cephalosporin monotherapy compared to cephalosporin combination therapy, while this advantage was less pronounced in females. Differences in immune and endocrine systems may partly explain this disparity. Men typically exhibited stronger, but less well-regulated, innate immune responses, such as enhanced TLR/NF-κB signaling and neutrophil activity. This robust inflammatory profile might render monotherapy sufficiently effective for pathogen clearance, while combination therapy could paradoxically worsen outcomes through excessive bacterial lysis and subsequent inflammatory cascades [42,43]. In contrast, estrogen in females enhanced neutrophil function, promoted Th2-mediated humoral immunity, and facilitated anti-inflammatory pathways like IL-10 signaling, potentially buffering the inflammatory cost of combination therapy [42]. Additionally, testosterone also downregulated Toll-like receptor expression and pro-inflammatory cytokine production, and reduced B-cell antibody responses, which might further attenuate the incremental benefit of multi-drug regimens in men [44]. Pharmacokinetic variations further contribute to these gender differences. Males generally exhibited higher glomerular filtration rates and distinct volume of distribution patterns [45], which might lead to suboptimal drug concentrations during combination therapy, increasing toxicity risks without commensurate antimicrobial benefits. Moreover, prescription patterns revealed potential gender biases. While one study reported no gender differences in antimicrobial use [46], a meta-analysis showed that women received more cephalosporin prescriptions than males [47], raising concerns about unequal dosing practices. This higher exposure could contribute to increased risk of antibiotic resistance in females, particularly when combined with evidence of gender-specific microbial profiles. Also, evidence suggested gender-specific variations in microbial profiles [48], which implied the need for tailored antibiotic approaches. These findings highlight the need for gender-tailored prophylactic strategies that integrate both biological and prescribing differences.

It is widely recognized that antibiotic prophylaxis could improve survival quality of elderly hip fracture patients. Our results demonstrated that participants with internal fixation acquired more benefits from cephalosporin monotherapy than from cephalosporin combination therapy. A study found a predominance of Gram-positive bacteria [49] in hip fracture surgeries. Cephalosporins, especially cefazolin, could cover most of the common orthopedic pathogens (S. aureus and coagulase-negative staphylococci), which might be sufficient [50]. Conversely, there are higher risks of Gram-negative infections (e.g., *Escherichia coli*) and biofilm formation on prostheses, where the spectrum of cephalosporin monotherapy might be insufficient [6]. Also, compared with hip replacement, internal fixation was associated with shorter operative times and less intraoperative bleeding [51]. Another study found that the type of surgery was related to prognostic outcomes for elderly patients [52]. These procedural differences could lead to more effective antibiotic prophylaxis with cephalosporin monotherapy for internal fixation, while simultaneously introducing greater unpredictability in prophylactic outcomes for patients with hip replacement. One study revealed higher rates of adverse outcomes when non-cefazolin antibiotics were used [53]. A preclinical exploratory analysis of patients receiving gentamicin-containing SAP combinations showed that they had a lower percentage of positive intraoperative cultures [49]. Therefore, this discrepancy may reflect unaddressed variables like regional resistance patterns, warranting further investigation into optimized dosing or adjunctive strategies for joint arthroplasty.

It should be noted that prognosis (e.g., mortality and infection risks) and antibiotic efficacy are profoundly influenced by host-related characteristics, including comorbidity burden, systemic inflammatory state, and frailty [54]. Our findings demonstrated that elderly patients with CCI ≥ 5 derived expected benefits from monotherapy, while extending this host-related framework to show that patients with multiple injuries or osteoporosis exhibited particular advantages with cephalosporin monotherapy, likely through reduced inflammatory stress and decreased toxicity accumulation, respectively.

Beyond conventional host factors, our study calculated SII as a novel inflammatory marker. SII reflects activation of the immune–coagulation–inflammation network and strongly correlates with patient prognosis through multiple pathways: CD40L-mediated endothelial injury, NETosis-induced organ damage, and lymphocyte exhaustion leading to post-septic immune paralysis [20]. A study obtained the optimal cut-off value of approximately 1752 on the fifth day after surgery using ROC/Youden to predict one-year mortality [27]. In our study, differential cut-off values suggested that the prognostic significance of SII varied according to both outcome severity and temporal trajectory, with more stringent thresholds required for predicting acute critical illness compared to longer-term mortality.

As highlighted, the host’s immunological and metabolic status critically determines infection severity, as it governs the ability of innate and adaptive immune responses to control bacterial colonization and regulate inflammation [55,56]. Comorbidity disrupted immune homeostasis, exacerbating infection susceptibility and complicating treatment responses, reducing survival quality in elderly hip fracture patients [57]. Multiple injuries entailed systemic inflammation and tissue hypoperfusion [54], potentially impairing antibiotic distribution and amplifying drug–drug interactions; thus, avoiding unnecessary combinations may reduce physiological stress and improve short-term outcomes. Comorbidities such as diabetes or osteoporosis might impair antibiotic efficacy, delay fracture healing, and increase susceptibility to infectious and non-infectious complications by altering tissue perfusion (e.g., microangiopathy, reducing drug delivery to infected sites) or promoting biofilm formation (e.g., hyperglycemia, enhancing bacterial adhesion to prosthetic materials) [58]. The use of antibiotics might alleviate the high risk of poor prognosis related to host-related factors. For instance, immunocompromised hosts, such as those with antineutrophil cytoplasmic antibody-associated vasculitis (AAV) and giant cell arteritis (GCA), had a higher risk of infection, but the use of antibiotics could help prevent some diseases, such as pneumonia [59]. Moreover, avoiding unnecessary combination therapy may reduce the risk of organ dysfunction triggered by drug toxicity, thereby preventing deterioration from non-infectious causes of death, which are particularly prevalent in frail patients with comorbidity.

Interestingly, we observed that the survival benefit of cephalosporin monotherapy was most evident in immunocompetent patients and similar across all SII subgroups, whereas this advantage was attenuated in immunosuppressed individuals. Also, minimal differences in infection risks were observed between cephalosporin monotherapy and combination therapy, which is consistent with prior research [60]. Some patients in the study were critically frail, and some had cardiovascular risk factors and comorbidity, including hypertension, heart failure, and myocardial infarction, which might reduce physiological reserve and contribute to poor antibiotic response. In extremely frail patients, the distribution volume of hydrophilic antibiotics, such as β-lactam, was reduced by 20–30% [61]. The phenomenon observed in our study might suggest that antibiotic efficacy depends on intact host immunity for synergistic pathogen clearance. In immunosuppressed or critically ill patients, compromised innate and adaptive immune responses may blunt the protective effect of monotherapy, highlighting the critical role of host immune competence in shaping prophylactic outcomes. Together, these results underscore the need for individualized prophylaxis strategies that account for both comorbidity burden and immunological status in elderly hip fracture patients.

The dose–response analysis revealed a U-shaped relationship for most antibiotic prophylaxis regimens, except for a linear association between non-drug use and 28-day mortality, likely due to small event sizes. In general, consistent with previous studies [14,62], we confirmed that an intermediate dose range (2 g to 3 g for cephalosporins) optimally reduced mortality and other outcomes, close to the gold standard for most of the cephalosporins made by the WHO [21]. The U-shaped curve underscored the risks of both insufficient and excessive dosing. Overdosing might induce nephrotoxicity, hepatotoxicity [36], or immune dysfunction through direct or indirect immunomodulation (e.g., IL-10 suppression) [63]. While the use of multiple agents could disturb host and institutional flora [64], increase bacterial resistance, and predispose patients to Clostridium difficile colitis [65], thereby elevating mortality risk. Conversely, underdosing may compromise pathogen eradication and drive antimicrobial resistance [66]. Notably, recent studies found heterogeneity in pharmacokinetic and pharmacodynamic profiles among patients, particularly those with hypoalbuminemia, critical illness, or organ dysfunction. Hypoalbuminemic patients required higher cephalosporin doses due to altered pharmacokinetics [67], highlighting the importance of individualized dosing. In frail multimorbid patients, the balance between efficacy and toxicity becomes precarious—higher doses may offset immune dysfunction yet concurrently exacerbate adverse effects, possibly contributing to the U-shaped mortality curve observed. Therefore, both antimicrobial-specific pharmacokinetic and pharmacodynamic properties and patient-specific factors should guide dosing. Advanced tools such as therapeutic drug monitoring, population pharmacokinetic modeling, and Bayesian forecasting may further individualize regimens to maximize therapeutic exposure while minimizing toxicity. Future clinical and mechanistic studies are warranted to clarify the underlying biology and establish precision dosing algorithms integrating patient characteristics, drug properties, and infection severity, ultimately refining prophylaxis strategies and improving outcomes.

Our study provides evidence to guide clinical practice in antibiotic prophylaxis for elderly hip fracture patients. The demonstrated long-term survival benefits of cephalosporin monotherapy, particularly evident at 90-day to 1-year follow-up, support its use as first-line prophylaxis in routine orthopedic surgery. These findings carry important implications for antimicrobial stewardship, suggesting that combination regimens should be reserved for specific high-risk cases such as confirmed MRSA infections, open fractures, or immunocompromised patients, given their similar protective benefits. Importantly, in real cases, proper antibiotic selection and dosing not only prevent infections but may also influence long-term survival outcomes. An exploration of consequences could prompt surgeons to evaluate their prophylactic protocols and favor appropriate doses as a balanced approach that maximizes patient outcomes while minimizing resistance development and healthcare costs. The study ultimately provides clinicians with an evidence-based framework to optimize antibiotic use in hip fracture surgery, benefiting both individual patients and broader antimicrobial stewardship efforts.

This study had several limitations. First, residual confounding may persist due to unmeasured factors [68]. We attempted to mitigate this by using IPTW and incorporating sensitivity analyses with broader inclusion criteria. Second, our study assessed all-cause mortality rather than cause-specific mortality due to the lack of death etiology data in MIMIC. Third, we were unable to assess the role of specific cephalosporin agents such as cefazolin, or the duration of surgery and prophylaxis, which might be another factor [69]. More studies are warranted to investigate the role of durations in elderly hip fracture patients. Finally, data on clinical decision-making (e.g., guideline adherence) were unavailable. Thus, further prospective randomized studies are needed to address these limitations.

Future research should aim to validate these findings through prospective, randomized controlled trials that minimize residual confounding and provide granular data on mortality causes. Such studies should also clarify the comparative effectiveness of different prophylactic regimens and define optimal prophylaxis duration using pharmacokinetic and pharmacodynamic modeling. Beyond regimen comparisons, future work should explore host-directed therapies to optimize personalized interventions, investigate biological mechanisms underlying outcome disparities through multi-omics approaches, and integrate these insights into risk-stratified clinical algorithms. Parallel evaluation of real-world cost-effectiveness and antimicrobial resistance trends will be essential to ensure that advances in perioperative prophylaxis translate into both improved patient outcomes and sustainable antimicrobial stewardship.

## 5. Conclusions

In conclusion, our study found relatively long-term survival benefits of cephalosporin monotherapy, especially for males, internal fixation patients, patients with CCI ≥ 5, and those with multiple injuries or osteoporosis, which could provide clinical insights and may provide a foundational basis for subsequent studies. Clinicians should cautiously select cephalosporin combination therapy for elderly hip fracture patients. More studies with larger sample sizes are needed to validate these findings and explore the possible cause-specific association between antibiotic prophylaxis and elderly patients’ prognosis.

## Figures and Tables

**Table 1:** Baseline characteristics of hip fracture patients after IPTW.

	All Patients (*n* = 4024.24)	Non-Users (*n* = 145.04)	Cephalosporin Monotherapy Users (*n* = 2590.05)	Non-Cephalosporin Users (*n* = 411.86)	Cephalosporin Combination Therapy Users (*n* = 877.30)	*p*-Value
**Age (years)**	80.00 ± 9.86	81.45 ± 9.85	79.92 ± 9.84	80.16 ± 9.50	79.92 ± 10.06	0.509
**Gender, *n* (%)**						0.916
Female	2771.4 (68.9)	102.9 (71.0)	1782.1 (68.8)	288.2 (70.0)	598.1 (68.2)	
Male	1252.8 (31.1)	42.1 (29.0)	807.9 (31.2)	123.6 (30.0)	279.2 (31.8)	
**Anchor year, *n* (%)**						0.999
2008–2010	1548.2 (38.5)	55.3 (38.1)	996.0 (38.5)	158.1 (38.4)	338.8 (38.6)	
2011–2013	877.3 (21.8)	28.7 (19.8)	565.6 (21.8)	90.0 (21.8)	193.2 (22.0)	
2014–2016	716.4 (17.8)	22.2 (15.3)	463.9 (17.9)	74.2 (18.0)	156.1 (17.8)	
2017–2019	495.7 (12.3)	22.7 (15.7)	316.2 (12.2)	51.1 (12.4)	105.7 (12.1)	
2020–2022	386.7 (9.6)	16.2 (11.2)	248.4 (9.6)	38.6 (9.4)	83.5 (9.5)	
**Race, *n* (%)**						0.999
White	3414.2 (84.8)	125.6 (86.6)	2190.7 (84.6)	355.3 (86.3)	742.6 (84.6)	
Asian	66.4 (1.7)	2.5 (1.7)	43.0 (1.7)	5.9 (1.4)	14.9 (1.7)	
Black	221.9 (5.5)	9.1 (6.3)	143.3 (5.5)	20.1 (4.9)	49.4 (5.6)	
Hispanic	67.0 (1.7)	1.3 (0.9)	45.6 (1.8)	6.4 (1.6)	13.7 (1.6)	
Other	254.6 (6.3)	6.4 (4.4)	167.4 (6.5)	24.1 (5.9)	56.7 (6.5)	
**Admission type, *n* (%)**						0.960
Emergence	2188.7 (54.4)	81.9 (56.5)	1412.3 (54.5)	223.6 (54.3)	470.9 (53.7)	
Elective	79.7 (2.0)	0.0 (0.0)	53.7 (2.1)	8.2 (2.0)	17.8 (2.0)	
Observation	880.0 (21.9)	35.0 (24.2)	567.7 (21.9)	90.0 (21.9)	187.2 (21.3)	
Same-day surgery	551.7 (13.7)	14.2 (9.8)	350.3 (13.5)	58.8 (14.3)	128.4 (14.6)	
Urgent	324.2 (8.1)	13.9 (9.6)	206.1 (8.0)	31.2 (7.6)	73.0 (8.3)	
**Total mortality, *n* (%)**	1422.7 (35.4)	53.4 (36.8)	875.2 (33.8)	142.3 (34.6)	351.7 (40.1)	0.039
**28-day mortality, *n* (%)**	198.7 (4.9)	3.0 (2.0)	100.4 (3.9)	24.4 (5.9)	70.9 (8.1)	<0.001
**90-day mortality, *n* (%)**	454.9 (11.3)	18.1 (12.5)	252.7 (9.8)	48.9 (11.9)	135.2 (15.4)	0.005
**180-day mortality, *n* (%)**	618.9 (15.4)	26.3 (18.2)	353.3 (13.6)	70.2 (17.0)	169.0 (19.3)	0.009
**1-year mortality, *n* (%)**	834.7 (20.7)	32.2 (22.2)	494.0 (19.1)	87.4 (21.2)	221.1 (25.2)	0.016
**Infection, *n* (%)**	226.3 (5.6)	0.8 (0.5)	149.9 (5.8)	22.7 (5.5)	52.9 (6.0)	0.088
**ICU admission, *n* (%)**	490.3 (12.2)	9.4 (6.5)	316.4 (12.2)	52.8 (12.8)	111.7 (12.7)	0.220
**Length of hospital stay (days)**	6.05 ± 4.32	4.78 ± 2.12	5.44 ± 3.36	6.53 ± 5.40	6.84 ± 5.74	0.369
**Charlson comorbidity index**	5.77 ± 2.24	5.85 ± 2.13	5.76 ± 2.29	5.77 ± 2.17	5.81 ± 2.15	0.937
**Osteoporosis, *n* (%)**	1200.2 (23.9)	54.6 (29.5)	763.7 (23.2)	143.1 (27.8)	238.7 (23.0)	0.096
**Multiple injuries, *n* (%)**	2254.6 (44.9)	79.0 (42.7)	1461.5 (44.5)	262.9 (51.1)	451.2 (43.5)	0.780
**Vital signs at presentation**						
BMI (kg/m^2^)	27.60 ± 15.70	26.26 ± 5.01	27.51 ± 17.90	27.02 ± 5.38	28.35 ± 12.93	0.054
Systolic blood pressure (mmHg)	131.91 ± 14.42	133.15 ± 13.39	131.95 ± 14.48	131.21 ± 14.97	131.92 ± 14.14	0.671
Diastolic blood pressure (mmHg)	73.03 ± 8.78	72.58 ± 8.11	73.02 ± 8.79	73.07 ± 9.10	73.08 ± 8.69	0.959
**Laboratory-based data**						
Red blood cell (109/L)	3.49 ± 0.63	3.49 ± 0.69	3.49 ± 0.62	3.47 ± 0.64	3.49 ± 0.61	0.971
White blood cell (109/L)	10.63 ± 7.05	10.52 ± 5.01	10.44 ± 4.23	12.14 ± 18.01	10.47 ± 4.24	0.844
Platelet (109/L)	212.04 ± 84.29	204.21 ± 73.59	212.09 ± 84.67	214.48 ± 87.36	212.06 ± 83.37	0.796
Hemoglobin (g/dL)	10.54 ± 1.79	10.49 ± 1.95	10.55 ± 1.80	10.50 ± 1.73	10.54 ± 1.76	0.963
Creatinine (mg/dL)	1.12 ± 0.93	1.14 ± 0.87	1.12 ± 0.92	1.14 ± 1.02	1.13 ± 0.92	0.983
BUN (mg/dL)	22.50 ± 12.88	23.20 ± 11.98	22.42 ± 12.88	22.45 ± 13.02	22.65 ± 12.96	0.928
Chloride (mmol/L)	102.56 ± 4.20	102.86 ± 3.93	102.54 ± 4.12	102.62 ± 4.62	102.57 ± 4.30	0.887
Bicarbonate (mmol/L)	25.11 ± 3.44	24.93 ± 3.71	25.14 ± 3.45	24.93 ± 3.43	25.12 ± 3.38	0.791
Potassium (mmol/L)	4.26 ±0.59	4.29 ± 0.55	4.26 ± 0.58	4.25 ± 0.62	4.25 ± 0.59	0.944
Sodium (mmol/L)	138.14 ± 3.77	138.67 ± 3.36	138.12 ± 3.68	138.05 ± 4.24	138.17 ± 3.85	0.428
Anion gap (mmol/L)	13.42 ± 3.14	13.46 ± 3.01	13.41 ± 3.22	13.36 ± 3.03	13.46 ± 2.97	0.962
Glucose (mg/dl)	135.60 ± 47.45	137.02 ± 49.18	135.41 ± 45.12	136.05 ± 53.09	135.73 ± 51.02	0.986
Lymphocyte count (109/L)	3.58 ± 5.23	1.06 ± 5.53	4.70 ± 3.84	4.03 ± 4.24	1.29 ± 1.62	0.249
Neutrophil count (109/L)	8.87 ± 4.62	10.14 ± 8.55	8.84 ± 4.03	7.97 ± 4.89	8.87 ± 4.96	0.571
**Treatment information, *n* (%)**						
Mechanical ventilation	434.2 (10.8)	7.8 (5.4)	281.6 (10.9)	47.7 (11.6)	97.1 (11.1)	0.226
Renal replacement therapy	1.5 (0.0)	0.0 (0.0)	0.0 (0.0)	0.0 (0.0)	1.5 (0.2)	0.167
**Surgery**						0.441
Internal fixation	2456.2 (61.0)	99.7 (68.7)	1579.9 (61.0)	246.0 (59.7)	530.6 (60.5)	
Hip replacement	1568.1 (39.0)	45.4 (31.3)	1010.1 (39.0)	165.9 (40.3)	346.7 (39.5)	
**Drug use, *n* (%)**						
Dopamine	15.4 (0.4)	0.0 (0.0)	11.8 (0.5)	0.9 (0.2)	2.8 (0.3)	0.725
Epinephrine	5.6 (0.1)	0.0 (0.0)	3.6 (0.1)	0.5 (0.1)	1.5 (0.2)	0.891
Furosemide	130.3 (3.2)	1.1 (0.8)	83.3 (3.2)	14.7 (3.6)	31.2 (3.6)	0.434
Norepinephrine	68.5 (1.7)	0.0 (0.0)	45.8 (1.8)	6.3 (1.5)	16.3 (1.9)	0.588
Phenylephrine	132.9 (3.3)	2.1 (1.5)	87.0 (3.4)	12.8 (3.1)	31.0 (3.5)	0.591
Immunosuppressant	452 (9.0)	20 (9.7)	204 (6.2)	67 (13.3)	161 (15.3)	0.049

**Table 2:** Primary outcomes in the post hoc pairwise comparisons.

Outcome	Non-Users (*n* = 145.04)	Cephalosporin Monotherapy Users (*n* = 2590.05)	Non-Cephalosporin Users (*n* = 411.86)	Cephalosporin Combination Therapy Users (*n* = 877.3)	All Patients (*n* = 4024.24)
**28** **-** **day mortality**					
No. of participants/No. at risk (%)	3.0/145.04 (2.00)	100.4/2590.05 (3.90)	24.4/411.86 (5.90)	70.9/877.3 (8.10)	198.7/4024.24 (4.90)
Rate (95% CI)―events/100 participant-day	0.07 (0.01–0.19)	0.14 (0.11–0.17)	0.22 (0.14–0.33)	0.31 (0.24–0.39)	0.18 (0.16–0.21)
Pairwise hazard ratio (95% CI)					
None		0.51 (0.15–1.74)	0.33 (0.09–1.19)	0.24 (0.07–0.78) *	
Cephalosporins			0.65 (0.34–1.24)	0.46 (0.28–0.75) *	
Other drugs				0.71 (0.39–1.29)	
Cephalosporins combined with other drugs					
**90** **-** **day mortality**					
No. of participants/No. at risk (%)	18.1/145.04 (12.5)	252.7/2590.05 (9.8)	48.9/411.86 (11.9)	135.2/877.3 (15.4)	454.9/4024.24 (11.30)
Rate (95% CI)―events/100 participant-day	0.13 (0.08–0.20)	0.11 (0.10–0.13)	0.15 (0.11–0.20)	0.20 (0.16–0.23)	0.13 (0.12–0.15)
Pairwise hazard ratio (95% CI)					
None		1.32 (0.51–3.38)	1.10 (0.40–2.98)	0.79 (0.31–2.04)	
Cephalosporins			0.83 (0.54–1.30)	0.60 (0.44–0.82) *	
Other drugs				0.72 (0.46–1.14)	
Cephalosporins combined with other drugs					
**180** **-** **day mortality**					
No. of participants/No. at risk (%)	26.3/145.04 (18.2)	353.3/2590.05 (13.6)	70.2/411.86 (17.0)	169.0/877.3 (19.3)	618.9/4024.24 (15.4)
Rate (95% CI)―events/100 participant-day	0.10 (0.06–0.14)	0.08 (0.07–0.09)	0.11 (0.09–0.14)	0.13 (0.11–0.15)	0.09 (0.08–0.10)
Pairwise hazard ratio (95% CI)					
None		1.37 (0.65–2.88)	1.09 (0.49–2.43)	0.92 (0.43–1.95)	
Cephalosporins			0.80 (0.55–1.16)	0.67 (0.51–0.87) *	
Other drugs				0.84 (0.57–1.24)	
Cephalosporins combined with other drugs					
**1** **-** **year mortality**					
No. of participants/No. at risk (%)	32.2/145.04 (22.2)	494.0/2590.05 (19.1)	87.4/411.86 (21.2)	221.1/877.3 (25.2)	834.7/4024.24 (20.7)
Rate (95% CI)―events/100 participant-day	0.06 (0.04–0.09)	0.06 (0.05–0.07)	0.07 (0.06–0.09)	0.06 (0.05–0.07)	0.09 (0.78–0.10)
Pairwise hazard ratio (95% CI)					
None		1.22 (0.63–2.33)	1.09 (0.54–2.21)	0.86 (0.44–1.67)	
Cephalosporins			0.90 (0.64–1.26)	0.71 (0.57–0.89) *	
Other drugs				0.79 (0.55–1.13)	
Cephalosporins combined with other drugs					

* Statistically significant according to the *p*-value adjusted by FDR.

**Table 3:** Secondary outcomes in the post hoc pairwise comparisons.

Outcome	Non-Users (45.04)	Cephalosporin Monotherapy Users (*n* = 2590.05)	Non-Cephalosporin Users (*n* = 411.86)	Cephalosporin Combination Therapy Users (*n* = 877.3)	All Patients (*n* = 4024.24)
**Infection**					
No. of participants/No. at risk (%)	0.8/145.04 (0.5)	149.9/2590.05 (5.8)	22.7/411.86 (5.5)	52.9/877.3 (6.0)	226.3/4024.24 (5.6)
Rate (95% CI)―events/100 participant-day	0.11 (0.003–0.64)	1.13 (0.96–1.33)	0.81 (0.53–1.26)	0.66 (0.50–0.87)	0.91 (0.80–1.04)
Odds ratio (95% CI)					
None		0.10 (0.01–1.38)	0.10 (0.01–1.36)	0.10 (0.01–1.33)	
Cephalosporins			0.94 (0.45–1.98)	0.98 (0.61–1.55)	
Other drugs				1.04 (0.53–2.02)	
Cephalosporins combined with other drugs					
**ICU admission**					
No. of participants/No. at risk (%)	9.4/145.04 (6.5)	316.4/2590.05 (12.2)	52.8/411.86 (12.8)	111.7/877.3 (12.7)	490.3/4024.24 (12.2)
Rate (95% CI)―events/100 participant-day	1.34 (0.61–2.52)	2.60 (2.33–2.90)	2.36 (1.77–3.08)	2.07 (1.71–2.49)	2.40 (2.19–2.61)
Odds ratio (95% CI)					
None		0.50 (0.26–0.96)	0.44 (0.21–0.96)	0.48 (0.25–0.91)	
Cephalosporins			0.89 (0.52–1.51)	0.96 (0.70–1.32)	
Other drugs				1.08 (0.64–1.81)	
Cephalosporins combined with other drugs					

**Table 4:** Primary outcomes in the post hoc pairwise comparisons after including patients aged 18 or above.

Outcome	Non-Users (*n* = 185.28)	Cephalosporin Monotherapy Users (*n* = 3290.05)	Non-Cephalosporin Users (*n* = 514.62)	Cephalosporin Combination Therapy Users (*n* = 1037.47)	All Patients (*n* = 5027.42)
**28** **-** **day mortality**					
No. of participants/no. at risk (%)	3.0/185.28 (1.60)	112.5/3290.05 (3.40)	25.1/514.62 (4.90)	72.3/1037.47 (7.00)	213.0/5027.42 (4.20)
Rate (95% CI)―events/100 participant-day	0.06 (0.12–0.17)	0.12 (0.10–0.15)	0.17 (0.11–0.26)	0.29 (0.19–0.31)	0.15 (0.13–0.17)
Pairwise hazard ratio (95% CI)					
None		0.42 (0.11–1.57)	0.27 (0.07–1.06)	0.19 (0.05–0.68) *	
Cephalosporins			0.66 (0.34–1.28)	0.45 (0.27–0.75) *	
Other drugs				0.68 (0.38–1.23)	
Cephalosporins combined with other drugs					
**90** **-** **day mortality**					
No. of participants/no. at risk (%)	19.0/185.28 (10.2)	279.0/3290.05 (8.5)	49.1/514.62 (9.5)	136.5/1037.47 (13.2)	483.5/5027.42 (9.60)
Rate (95% CI)―events/100 participant-day	0.11 (0.07–0.18)	0.02 (0.01–0.03)	0.11 (0.08–0.14)	0.15 (0.12–0.17)	0.11 (0.10–0.12)
Pairwise hazard ratio (95% CI)					
None		1.24 (0.55–2.76)	0.99 (0.42–2.33)	0.68 (0.31–1.53)	
Cephalosporins			0.80 (0.52–1.24)	0.55 (0.40–0.76) *	
Other drugs				0.69 (0.45–1.06)	
Cephalosporins combined with other drugs					
**180** **-** **day mortality**					
No. of participants/no. at risk (%)	29.0/185.28 (15.7)	392.0/3290.05 (11.9)	71.9/514.62 (14.0)	171.7/1037.47 (16.6)	664.6/5027.42 (13.2)
Rate (95% CI)―events/100 participant-day	0.09 (0.06–0.12)	0.07 (0.06–0.08)	0.08 (0.06–0.10)	0.09 (0.08–0.11)	0.07 (0.06–0.08)
Pairwise hazard ratio (95% CI)					
None		1.33 (0.71–2.50)	1.03 (0.52–2.03)	0.80 (0.42–1.50)	
Cephalosporins			0.77 (0.53–1.11)	0.60 (0.46–0.78) *	
Other drugs				1.13 (0.85–1.50)	
Cephalosporins combined with other drugs					
**1** **-** **year mortality**					
No. of participants/no. at risk (%)	41.8/185.28 (22.6)	548.8/3290.05 (16.7)	91.0/514.62 (17.7)	226.8/1037.47 (21.9)	908.4/5027.42 (18.1)
Rate (95% CI)―events/100 participant-day	0.06 (0.04–0.08)	0.05 (0.04–0.06)	0.05 (0.04–0.06)	0.06 (0.05–0.07)	0.05 (0.04–0.053)
Pairwise hazard ratio (95% CI)					
None		1.44 (0.83–2.52)	1.18 (0.64–2.18)	0.90 (0.51–1.59)	
Cephalosporins			0.82 (0.60–1.13)	0.63 (0.50–0.78) *	
Other drugs				0.76 (0.54–1.07)	
Cephalosporins combined with other drugs					

* Statistically significant according to the *p*-value adjusted by FDR.

**Table 5:** Secondary outcomes in the post hoc pairwise comparisons after including patients aged 18 or above.

Outcome	Non-Users (*n* = 185.28)	Cephalosporin Monotherapy Users (*n* = 3290.05)	Non-Cephalosporin Users (*n* = 514.62)	Cephalosporin Combination Therapy Users (*n* = 1037.47)	All Patients (*n* = 5027.42)
**Infection**					
No. of participants/No. at risk (%)	1.9/185.28 (1.0)	209.5/3290.05 (6.4)	36.8/514.62 (7.1)	68.4/1037.47 (6.6)	316.5/5027.42 (6.3)
Rate (95% CI)―events/100 participant-day	0.17 (0.02–0.64)	1.31 (1.15–1.51)	1.31 (0.87–1.69)	0.90 (0.69–1.13)	1.14 (1.02–1.27)
Odds ratio (95% CI)					
None		2.94 (0.41–21.38)	8.97 (1.21–66.42)	13.72 (1.90–99.31)	
Cephalosporins			1.14 (0.66–1.99)	1.01 (0.74–1.38)	
Other drugs				0.87 (0.55–1.38)	
Cephalosporins combined with other drugs					
**ICU admission**					
No. of participants/No. at risk (%)	17.4/185.28 (9.4)	433.0/3290.05 (13.2)	72.2 /514.62 (14.0)	146.9/1037.47 (14.2)	669.5/5027.42 (13.3)
Rate (95% CI)―events/100 participant-day	2.01 (1.15–3.13)	2.90 (2.64–3.19)	2.65 (2.07–3.31)	2.32 (1.96–2.72)	2.69 (2.62–3.03)
Odds ratio (95% CI)					
None		0.90 (0.50–1.64)	1.38 (0.82–2.34)	2.48 (1.56–3.96)	
Cephalosporins			1.15 (0.80–1.66)	1.08 (0.86–1.35)	
Other drugs				0.95 (0.66–1.35)	
Cephalosporins combined with other drugs					

## Data Availability

The datasets presented in this study can be found in online repositories. The names of the repository/repositories and accession number(s) can be found below. All data and materials are available at https://mimic.mit.edu/. The generated data used for the study can be found in the Supplementary Materials.

## Supplementary Materials

The following supporting information can be downloaded at https://www.mdpi.com/article/10.3390/jcm14176086/s1: Supplementary Tables S1–S6: Supplementary Table S1: Generated data for the analysis of the study; Supplementary Table S2: Baseline characteristics of elderly hip fracture patients before IPTW; Supplementary Table S3: Descriptive presentation of primary outcomes stratified by subgroups (except SII); Supplementary Table S4: Descriptive presentation of secondary outcomes stratified by subgroups (except SII); Supplementary Table S5: Descriptive presentation of primary and secondary outcomes stratified by SII; Supplementary Table S6: The interaction *p*-value of four drug groups and subgroups for both primary and secondary outcomes. Supplementary Figures S1–S5; Supplementary Figure S3: Associations between cephalosporin use and 1-year mortality stratified by subgroups; Supplementary Figure S4: Associations between cephalosporin use and ICU admission risk stratified by subgroups; Supplementary Figure S5: Associations between cephalosporin use and infection risk stratified by subgroups.
