# Supplementary material for: The Long-Term Efficacy of Cephalosporin in Elderly Hip Fracture Patients: A Comprehensive Analysis
Source: J Clin Med. 2025 Aug 28;14(17):6086. doi: 10.3390/jcm14176086 (PMC12429005; doi:10.3390/jcm14176086)
Supplement: Supplementary file 1 [file jcm-14-06086-s001.zip › Supplementary Table S2.pdf]

Supplementary Table S2: Baseline characteristics of elderly hip fracture patients before IPTW.

	All patients (n=4044)	Non-users (n=166)	Cephalosporin monotherapy users (n=2589)	Non-cephalosporin users (n=403)	Cephalosporin combination therapy users (n=886)	*P*-value
**Age (years)**	79.87±9.86	81.48±9.82	79.47±9.95	80.00±9.36	80.69±9.78	0.052
**Gender, n (%)**						0.001
Female	2776 (68.6)	113 (68.1)	1784 (68.9)	305 (75.7)	574 (64.8)	
Male	1268 (31.4)	53 (31.9)	805 (31.1)	98 (24.3)	312 (35.2)	
**Anchor year, n (%)**						<0.001
2008-2010	1549 (38.3)	46 (27.7)	943 (36.4)	200 (49.6)	360 (40.6)	
2011-2013	889 (22.0)	31 (18.7)	565 (21.8)	95 (23.6)	198 (22.3)	
2014-2016	707 (17.5)	23 (13.9)	450 (17.4)	61 (15.1)	173 (19.5)	
2017-2019	498 (12.3)	37 (22.3)	343 (13.2)	33 (8.2)	85 (9.6)	
2020-2022	401 (9.9)	29 (17.5)	288 (11.1)	14 (3.5)	70 (7.9)	
**Race, n (%)**						0.005
White	3431 (84.8)	139 (83.7)	2216 (85.6)	336 (83.4)	740 (83.5)	
Asian	68 (1.7)	5 (3.0)	47 (1.8)	7 (1.7)	9 (1.0)	
Black	219 (5.4)	7 (4.2)	153 (5.9)	22 (5.5)	37 (4.2)	
Hispanic	59 (1.5)	3 (1.8)	29 (1.1)	7 (1.7)	20 (2.3)	
Others	267 (6.6)	12 (7.2)	144 (5.6)	31 (7.7)	80 (9.0)	
**Admission type, n (%)**						<0.001
Emergence	2210 (54.6)	91 (54.8)	1319 (50.9)	259 (64.3)	541 (61.1)	
Elective	79 (2.0)	0 (0.0)	57 (2.2)	5 (1.2)	17 (1.9)	
Observation	883 (21.8)	58 (34.9)	609 (23.5)	60 (14.9)	156 (17.6)	
Surgical same day	545 (13.5)	4 (2.4)	418 (16.1)	40 (9.9)	83 (9.4)	
Urgent	327 (8.1)	13 (7.8)	186 (7.2)	39 (9.7)	89 (10.0)	
**Total mortality, n (%)**	1449 (35.8)	57 (34.3)	802 (31.0)	167 (41.4)	423 (47.7)	<0.001

**28-day mortality, n (%)**	215 (5.3)	6 (3.6)	72 (2.8)	31 (7.7)	106 (12.0)	<0.001
**90-day mortality, n (%)**	470 (11.6)	21 (12.7)	209 (8.1)	58 (14.4)	182 (20.5)	<0.001
**180-day mortality, n (%)**	634 (15.7)	28 (16.9)	302 (11.7)	81 (20.1)	223 (25.2)	<0.001
**1-year mortality, n (%)**	856 (21.2)	35 (21.1)	438 (16.9)	98 (24.3)	285 (32.2)	<0.001
**Infection, n (%)**	242 (6.0)	1 (0.6)	55 (2.1)	33 (8.2)	153 (17.3)	<0.001
**ICU admission, n (%)**	492 (12.2)	30 (18.1)	163 (6.3)	59 (14.6)	240 (27.1)	<0.001
**Length of hospital, (days)**	6.14±4.76	5.27±2.77	5.11±2.97	6.78±6.15	9.02±6.89	<0.001
**Charlson comorbidity index**	5.74±2.23	5.92±2.15	5.49±2.13	6.08±2.34	6.29±2.35	<0.001
**Osteoporosis, n (%)**	1199 (23.8)	55 (26.6)	750 (22.8)	148 (29.5)	246 (23.3)	0.009
**Multiple injuries, n (%)**	2240 (44.4)	102 (49.3)	1411 (43.0)	243 (48.4)	484 (45.9)	0.03
**Vital signs at presentation**						
BMI (kg/m2)	27.46±14.69	26.55±5.11	27.40±17.08	27.09±5.15	27.96±10.72	0.577
Systolic blood pressure (mmHg)	131.90±14.44	131.59±13.62	132.25±14.57	130.62±14.36	131.51±14.24	0.141
Diastolic blood pressure (mmHg)	73.04±8.82	71.55±8.24	73.37±8.88	72.68±9.02	72.51±8.60	0.007
**Laboratory-based data**						
Red blood cell (109/L)	3.50±0.63	3.41±0.68	3.51±0.62	3.50±0.63	3.49±0.63	0.243
White blood cell (109/L)	10.47±5.02	9.81±4.08	10.40±4.18	10.19±9.01	10.93±4.87	0.007
Platelet (109/L)	212.49±83.66	185.37±63.42	211.87±80.75	214.73±87.35	218.35±92.28	<0.001
Hemoglobin (g/dl)	10.57±1.80	10.32±1.96	10.62±1.80	10.53±1.71	10.47±1.79	0.036
Creatinine (mg/dl)	1.10±0.88	1.31±1.33	1.06±0.80	1.12±0.96	1.18±0.93	<0.001
BUN (mg/dl)	22.26±12.92	24.60±13.19	21.31±11.94	22.61±14.06	24.43±14.66	<0.001
Chloride (mmol/L)	102.54±4.21	103.05±4.17	102.46±4.00	102.68±4.48	102.61±4.66	0.254
Bicarbonate (mmol/L)	25.10±3.40	24.61±3.59	25.08±3.28	25.52±3.46	25.05±3.67	0.025

Potassium (mmol/L)	4.25±0.58	4.32±0.55	4.23±0.57	4.27±0.64	4.27±0.61	0.092
Sodium (mmol/L)	138.10±3.78	138.59±3.36	138.02±3.67	138.03±4.06	138.27±4.01	0.119
Anion gap (mmol/L)	13.39±3.09	13.21±3.26	13.26±3.09	13.35±3.05	13.82±3.04	<0.001
Glucose (mg/dl)	135.12±47.52	133.43±43.25	134.71±45.39	135.78±53.74	136.34±51.25	0.786
Lymphocyte count (109/L)	3.46±5.44	1.12±0.52	4.90±7.90	2.34±6.37	1.18±1.32	0.371
Neutrophil count (109/L)	9.10±4.80	8.01±5.70	8.91±4.07	8.10±5.31	9.95±5.72	0.013
**Treatment information, n (%)**						
Mechanical ventilation	432 (10.7)	25 (15.1)	135 (5.2)	53 (13.2)	219 (24.7)	<0.001
Renal replacement therapy	7 (0.2)	0 (0.0)	0 (0.0)	0 (0.0)	7 (0.8)	<0.001
Surgery						<0.001
Internal fixation	2457 (60.8)	128 (77.1)	1523 (58.8)	265 (65.8)	541 (61.1)	
Hip replacement	1587 (39.2)	38 (22.9)	1066 (41.2)	138 (34.2)	345 (38.9)	
**Drug use, n (%)**						
Dopamine	11 (0.3)	0 (0.0)	3 (0.1)	2 (0.5)	6 (0.7)	0.031
Epinephrine	7 (0.2)	0 (0.0)	2 (0.1)	2 (0.5)	3 (0.3)	0.138
Furosemide	144 (3.6)	3 (1.8)	33 (1.3)	19 (4.7)	89 (10.0)	<0.001
Norepinephrine	75 (1.9)	0 (0.0)	14 (0.5)	8 (2.0)	53 (6.0)	<0.001
Phenylephrine	132 (3.3)	5 (3.0)	38 (1.5)	18 (4.5)	71 (8.0)	<0.001
Immunosuppressant	452 (9.0)	20 (9.7)	204 (6.2)	67 (13.3)	161 (15.3)	<0.001
