# Supplementary material for: The Long-Term Efficacy of Cephalosporin in Elderly Hip Fracture Patients: A Comprehensive Analysis
Source: J Clin Med. 2025 Aug 28;14(17):6086. doi: 10.3390/jcm14176086 (PMC12429005; doi:10.3390/jcm14176086)
Supplement: Supplementary file 1 [file jcm-14-06086-s001.zip › Supplementary Table S3.pdf]

Supplementary Table S3: Descriptive presentation of primary outcomes stratified by subgroups (except SII).

			28-day mortality		90-day mortality		180-day mortality		1-year mortality	
**Subgroups**	Category	No. at risk	No. of events	Rates[1]	No. of events	Rates[1]	No. of events	Rates[1]	No. of events	Rates[1]
**Gender**										
Male	Group1[2]	43.54	0.7	0.07 (0.01-0.38)	10.1	0.23 (0.11-0.42)	12.0	0.15 (0.08-0.26)	12.9	0.08 (0.05-0.14)
	Group2[3]	800.1	32.5	0.15 (0.10-0.21)	92.2	0.13 (0.11-0.16)	137.5	0.10 (0.09-0.12)	188.1	0.07 (0.06-0.09)
	Group3[4]	108.74	7.6	031 (0.13-0.61)	12.4	0.16 (0.08-0.27)	19.5	0.14 (0.09-0.22)	24.1	0.09 (0.06-0.13)
	Group4[5]	307.1	33.9	0.43 (0.29-0.59)	66.2	0.29 (0.22-0.37)	80.7	0.19 (0.15-0.24)	101.2	0.13 (0.10-0.16)
Female	Group1[2]	101.97	1.0	0.03 (0.008-0.18)	9.4	0.09 (0.04-0.18)	14.6	0.08 (0.05-0.13)	20.0	0.06 (0.03-0.09)
	Group2[3]	1779.37	64.9	0.13 (0.10-0.17)	163.5	0.11 (0.09-0.12)	219.6	0.07 (0.06-0.08)	309.3	0.05 (0.04-0.06)
	Group3[4]	312.83	15.7	0.19 (0.11-0.31)	40.1	0.16 (0.11-0.22)	53.9	0.11 (0.08-0.15)	69.1	0.07 (0.06-0.09)
	Group4[5]	568.36	35.9	0.24 (0.17-0.33)	70.3	0.15 (0.12-0.19)	90.2	0.10 (0.08-0.13)	120.6	0.07 (0.06-0.08)
**Surgery**										
Internal fixation	Group1[2]	120.73	2.9	0.09 (0.02-0.25)	10.7	0.10 (0.05-0.18)	18.5	0.09 (0.06-0.14)	25.5	0.07 (0.04-0.10)
	Group2[3]	1534.91	64.3	0.15 (0.12-0.19)	161.7	0.12 (0.10-0.14)	225.3	0.09 (0.08-0.10)	313.7	0.06 (0.05-0.07)
	Group3[4]	272.9	22.3	0.31 (0.20-0.47)	44.4	0.21 (0.15-0.28)	61.7	0.15 (0.12-0.20)	73.3	0.09 (0.07-0.12)
	Group4[5]	534.59	54.0	0.38 (0.29-0.50)	98.3	0.24 (0.19-0.29)	124.8	0.16 (0.13-0.19)	154.4	0.11 (0.09-0.12)
Hip replacement	Group1[2]	32.01	0.1	/	15.2	0.47 (0.26-0.78)	16.9	0.28 (0.16-0.45)	17.0	0.15 (0.08-0.23)
	Group2[3]	1057.73	40.1	0.14 (0.10-0.19)	95.5	0.10 (0.08-0.13)	130.9	0.07 (0.06-0.09)	179.3	0.05 (0.04-0.06)
	Group3[4]	152.34	3.1	0.08 (0.02-0.23)	9.2	0.08 (0.04-0.14)	15.3	0.07 (0.04-0.11)	21.2	0.05 (0.03-0.07)
	Group4[5]	341.02	19.2	0.21 (0.12-0.32)	41.3	0.15 (0.11-0.20)	48.9	0.09 (0.08-0.12)	72.3	0.07 (0.06-0.09)
**CCI[6]**										
<5	Group1[2]	40.97	0.0	/	0.6	0.02 (0.005-0.12)	2.1	0.02 (0.003-0.08)	3.0	0.02 (0.003-0.08)
	Group2[3]	1040.41	3.0	0.01 (0.002-0.03)	21.0	0.02 (0.01-0.03)	40.6	0.02 (0.01-0.03)	57.2	0.02 (0.01-0.022)

	Group3[4]	124.7	1.5	0.10 (0.007-0.21)	7.2	0.06 (0.03-0.13)	9.7	0.05 (0.02-0.08)	10.3	0.02 (0.01-0.04)
	Group4[5]	221.27	10.6	0.19 (0.09-0.33)	14.4	0.08 (0.04-0.17)	15.3	0.04 (0.02-0.07)	23.5	0.03 (0.02-0.05)
≥5	Group1[2]	96	3.3	0.10 (0.02-0.28)	17.0	0.18 (0.11-0.29)	20.3	0.10 (0.07-0.18)	25.1	0.08 (0.05-0.11)
	Group2[3]	1549.32	94.2	0.22 (0.18-0.27)	216.0	0.17 (0.14-0.19)	294.8	0.12 (0.10-0.13)	408.4	0.09 (0.08-0.10)
	Group3[4]	282.66	26.1	0.35 (0.23-0.52)	48.7	0.22 (0.17-0.30)	66.9	0.16 (0.13-0.21)	87.0	0.11 (0.09-0.14)
	Group4[5]	663.72	70.0	0.40 (0.31-0.51)	139.0	0.28 (0.23-0.33)	178.1	0.19 (0.16-0.22)	228.7	0.13 (0.11-0.15)
**Multiple injuries**										
Yes	Group1[2]	76.87	0.0	/	8	0.11 (0.05-0.22)	12.5	0.10 (0.05-0.17)	15.3	0.06 (0.03-0.09)
	Group2[3]	1127.1	26.2	0.08 (0.05-0.12)	99.2	0.10 (0.08-0.12)	140.6	0.07 (0.06-0.09)	200.3	0.04 (0.03-0.04)
	Group3[4]	193.61	15.9	0.31 (0.17-0.50)	23.9	0.15 (0.10-0.22)	34.3	0.11 (0.08-0.15)	44.5	0.06 (0.04-0.07)
	Group4[5]	388.6	31.6	0.31 (0.21-0.44)	55.5	0.18 (0.14-0.23)	75.5	0.13 (0.10-0.16)	95.7	0.06 (0.05-0.08)
No	Group1[2]	70.95	2.5	0.13 (0.03-0.39)	14.6	0.22 (0.12-0.36)	15.8	0.12 (0.07-0.19)	19.2	0.08 (0.05-0.12)
	Group2[3]	1468.62	85.6	0.21 (0.17-0.26)	157.4	0.13 (0.11-0.15)	211.2	0.09 (0.08-0.10)	287.3	0.06 (0.05-0.07)
	Group3[4]	218.2	12.6	0.23 (0.12-0.40)	36.1	0.22 (0.15-0.30)	50.3	0.16 (0.12-0.21)	57.7	0.10 (0.07-0.12)
	Group4[5]	488.85	44.2	0.34 (0.25-0.46)	89.8	0.24 (0.19-0.29)	109.1	0.15 (0.13-0.19)	141.8	0.11 (0.09-0.13)
**Osteoporosis**										
Yes	Group1[2]	48.27	0.0	/	3.1	0.06 (0.01-0.18)	6.5	0.08 (0.03-0.16)	7.3	0.04 (0.02-0.08)
	Group2[3]	715.9	15.2	0.08 (0.04-0.12)	52	0.08 (0.06-0.11)	73.9	0.06 (0.05-0.08)	111.1	0.05 (0.04-0.06)
	Group3[4]	132.88	6	0.17 (0.06-0.36)	11	0.10 (0.05-0.17)	20.5	0.10 (0.06-0.15)	32.7	0.08 (0.05-0.11)
	Group4[5]	224.7	12.9	0.21 (0.11-0.37)	27.5	0.15 (0.10-0.22)	33.7	0.10 (0.07-0.13)	43.3	0.06 (0.04-0.08)
No	Group1[2]	95.3	3.3	0.10 (0.02-0.29)	20.5	0.23 (0.14-0.35)	24.2	0.14 (0.09-0.20)	30.4	0.09 (0.06-0.13)
	Group2[3]	1878.58	86.1	0.17 (0.13-0.21)	198.2	0.12 (0.11-0.14)	271.7	0.09 (0.08-0.10)	367.6	0.06 (0.05-0.07)
	Group3[4]	279.02	18.2	0.25 (0.15-0.40)	40.6	0.19 (0.14-0.26)	51.3	0.13 (0.09-0.16)	59.7	0.08 (0.06-0.10)
	Group4[5]	654.57	61.4	0.36 (0.27-0.46)	118.6	0.24 (0.20-0.28)	150.1	0.16 (0.13-0.19)	195.5	0.11 (0.10-0.13)
**Immunosupp**										

**ressant**										
Yes	Group1[2]	18.14	0.5	0.21 (0.01-1.16)	0.5	0.07 (0.009-0.38)	9.6	0.35 (0.17-0.64)	10.3	0.18 (0.09-0.33)
	Group2[3]	172.27	14.9	0.30 (0.17-0.50)	28.3	0.18 (0.12-0.27)	36	0.12 (0.09-0.17)	44.9	0.08 (0.06-0.11)
	Group3[4]	52.81	2.8	0.20 (0.04-0.59)	14.9	0.35 (0.20-0.57)	18.2	0.23 (0.13-0.36)	24.4	0.16 (0.10-0.24)
	Group4[5]	126.51	8.7	0.26 (0.12-0.48)	19.8	0.19 (0.12-0.30)	23.8	0.12 (0.08-0.18)	32.4	0.09 (0.06-0.12)
No	Group1[2]	128.91	1.3	0.02 (0.002-0.14)	19	0.15 (0.09-0.24)	24.3	0.10 (0.05-0.15)	30.7	0.07 (0.05-0.10)
	Group2[3]	2405.11	79.4	0.12 (0.09-0.15)	211.8	0.10 (0.09-0.12)	298.7	0.07 (0.06-0.08)	424.4	0.05 (0.04-0.06)
	Group3[4]	360.59	20	0.21 (0.13-0.33)	38.4	0.13 (0.09-0.18)	56.7	0.10 (0.07-0.13)	75.8	0.07 (0.06-0.09)
	Group4[5]	752.55	66.5	0.34 (0.26-0.43)	125.1	0.21 (0.18-0.25)	160.8	0.15 (0.12-0.17)	208	0.10 (0.09-0.11)

[1] Rates/100 participant-day (95%CI)

[2] Non-users

[3] Cephalosporin monotherapy

[4] Non-cephalosporin users

[5] Cephalosporin combination therapy users

[6] Charlson comorbidity index
