# Supplementary material for: The Long-Term Efficacy of Cephalosporin in Elderly Hip Fracture Patients: A Comprehensive Analysis
Source: J Clin Med. 2025 Aug 28;14(17):6086. doi: 10.3390/jcm14176086 (PMC12429005; doi:10.3390/jcm14176086)
Supplement: Supplementary file 1 [file jcm-14-06086-s001.zip › Supplementary Table S4.pdf]

Supplementary Table S4: Descriptive presentation of secondary outcomes stratified by subgroups (except SII).

			Infection		ICU admission	
**Subgroups**	Category	No. at risk	No. of events	Rates[1]	No. of events	Rates[1]
**Gender**						
Male	Group1[2]	43.54	0.0	/	1.8	0.80 (0.10-2.85)
	Group2[3]	800.1	55.1	1.33 (1.00-1.73)	113.3	2.99 (2.47-3.59)
	Group3[4]	108.74	5.3	0.59 (0.19-1.37)	10.5	0.29 (0.15-0.52)
	Group4[5]	307.1	24.8	0.73 (0.47-1.08)	45.4	7.06 (5.20-9.34)
Female	Group1[2]	101.97	0.6	0.18 (0.005-1.02)	6.4	1.42 (0.52-3.05)
	Group2[3]	1779.37	78.7	0.87 (0.69-1.08)	192.5	2.31 (2.00-2.65)
	Group3[4]	312.83	14.7	0.80 (0.45-1.31)	40.6	2.55 (1.84-3.44)
	Group4[5]	568.36	28.5	0.63 (0.42-0.91)	63.6	1.99 (1.54-2.54)
**Surgery**						
Internal fixation	Group1[2]	120.73	1.5	0.30 (0.04-1.09)	9.2	1.79 (0.82-3.38)
	Group2[3]	1534.91	110.7	1.38 (1.14-1.66)	218.4	3.00 (2.62-3.42)
	Group3[4]	272.9	14.9	0.82 (0.46-1.34)	38.4	2.56 (1.82-3.50)
	Group4[5]	534.59	38.5	0.78 (0.56-1.07)	72.6	2.27 (1.78-2.84)
Hip replacement	Group1[2]	32.01	0.0	/	0.5	0.58 (0.01-3.20)
	Group2[3]	1057.73	43.2	0.83 (0.60-1.11)	98.3	2.01 (1.64-2.45)
	Group3[4]	152.34	8.6	1.01 (0.46-1.90)	14.5	1.97 (1.11-3.23)
	Group4[5]	341.02	15.9	0.53 (0.30-0.86)	37.9	1.74 (1.23-2.38)
**CCI[6]**						
<5	Group1[2]	40.97	0.8	0.36 (0.09-1.99)	1.5	0.73 (1.21-2.38)

	Group2[3]	1040.41	20.9	0.46 (0.29-0.71)	60.0	1.39 (1.06-1.79)
	Group3[4]	124.7	0.6	0.15 (0.04-0.82)	1.4	0.16 (0.004-0.87)
	Group4[5]	221.27	4.2	0.28 (0.08-0.70)	12.6	1.28 (0.68-2.17)
≥5	Group1[2]	96	0.0	/	7.8	1.71 (0.74-3.33)
	Group2[3]	1549.32	120.2	1.38 (1.14-1.65)	247.1	3.16 (2.78-3.57)
	Group3[4]	282.66	17.9	0.72 (0.43-1.13)	45.6	2.86 (2.10-3.79)
	Group4[5]	663.72	56.2	0.86 (0.65-1.11)	107.7	2.46 (2.02-2.97)
**Multiple injuries**						
Yes	Group1[2]	76.87	1.2	0.45 (0.05-1.62)	5.7	1.84 (0.68-3.96)
	Group2[3]	1127.1	68	1.12 (0.87-1.41)	161.6	2.99 (2.55-3.48)
	Group3[4]	193.61	10	0.73 (0.35-1.34)	28.3	2.60 (1.73-3.74)
	Group4[5]	388.6	26	0.70 (0.46-1.02)	56.8	2.40 (1.83-3.10)
No	Group1[2]	70.95	0.0	/	4.1	1.15 (0.31-2.91)
	Group2[3]	1468.62	84.5	1.19 (0.95-1.47)	162.2	2.41 (2.06-2.81)
	Group3[4]	218.2	8.8	0.66 (0.30-1.24)	19.2	1.63 (0.98-2.53)
	Group4[5]	488.85	29.2	0.68 (0.46-0.98)	56.6	1.88 (1.43-2.43)
**Osteoporosis**						
Yes	Group1[2]	48.27	0.0	/	1.9	0.96 (0.12-3.43)
	Group2[3]	715.9	21.9	0.60 (0.38-0.91)	76.8	2.28 (1.80-2.84)
	Group3[4]	132.88	3.7	0.53 (0.14-1.35)	16.2	2.51 (1.44-4.04)
	Group4[5]	224.7	9.1	0.49 (0.23-0.93)	22.9	1.61 (1.02-2.41)
No	Group1[2]	95.3	0.9	0.16 (0.00-0.91)	7	1.50 (0.60-3.06)
	Group2[3]	1878.58	128.4	1.34 (1.12-1.59)	247.9	2.83 (2.49-3.20)

	Group3[4]	279.02	16	0.81 (0.46-1.31)	32.1	1.99 (1.37-2.80)
	Group4[5]	654.57	46.9	0.76 (0.56-1.01)	87.5	2.21 (1.78-2.72)
**Immunosuppressant**						
Yes	Group1[2]	18.14	0.0	/	0.0	/
	Group2[3]	172.27	8.1	0.71 (0.31-1.40)	26.3	2.50 (1.64-3.63)
	Group3[4]	52.81	5.1	0.88 (0.29-2.04)	6.4	1.43 (0.53-3.09)
	Group4[5]	126.51	15.6	1.03 (0.59-1.66)	24.9	2.73 (1.77-4.00)
No	Group1[2]	128.91	0.9	0.13 (0.007-0.70)	8.8	1.48 (0.68-2.78)
	Group2[3]	2405.11	121.3	1.00 (0.83-1.19)	276.7	2.50 (2.21-2.80)
	Group3[4]	360.59	13.1	0.60 (0.32-1.02)	46.4	2.52 (1.85-3.35)
	Group4[5]	752.55	42.8	0.67 (0.48-0.90)	88.8	1.98 (1.60-2.44)

[1] Rates/100 participant-day (95%CI)

[2] Non-users

[3] Cephalosporin monotherapy

[4] Non-cephalosporin users

[5] Cephalosporin combination therapy users

[6] Charlson comorbidity index
