# Supplementary material for: The Long-Term Efficacy of Cephalosporin in Elderly Hip Fracture Patients: A Comprehensive Analysis
Source: J Clin Med. 2025 Aug 28;14(17):6086. doi: 10.3390/jcm14176086 (PMC12429005; doi:10.3390/jcm14176086)
Supplement: Supplementary file 1 [file jcm-14-06086-s001.zip › Supplementary Table S5.pdf]

Supplementary Table S5: Descriptive presentation of primary and secondary outcomes stratified by SII.

**Outcomes**	Group1[1]		Group2[2]		Group3[3]		Group4[4]	
	NR/NE[5]	Rates[6]	NR/NE[5]	Rates[6]	NR/NE[5]	Rates[6]	NR/NE[5]	Rates[6]
**28-day mortality**								
≥1310.1	11.61/0.5	0.19 (0.004-0.13)	227.36/20.4	0.29 (0.17-0.44)	0.0/22	/	118.98/19.2	0.53 (0.32-0.82)
<1310.1	3.89/0.0	/	166.14/15.2	0.28 (0.16-0.46)	23.29/9.1	1.16 (0.53-2.20)	68.19/10.9	0.50 (0.25-0.89)
**90-day mortality**								
≥2077.5	4.47/0.0	/	118.5/22.1	0.18 (0.11-0.28)	8.89/1.0	0.07 (0.007-0.31)	80.23/25.1	0.37 (0.24-0.54)
<2077.5	24.0/2.4	0.10 (0.001-0.35)	280.17/40.1	0.15 (0.11-0.21)	46.11/9.3	0.27 (0.12-0.50)	107.04/28.0	0.28 (0.18-0.41)
**180-day mortality**								
≥2077.5	4.47/0.0	/	118.5/28.1	0.12(0.08-0.18)	8.89/1.3	0.07 (0.008-0.26)	80.23/30.2	0.24 (0.16-0.34)
<2077.5	24.0/2.4	0.05 (0.006-0.19)	280.17/46.8	0.09 (0.06-0.13)	46.11/10.9	0.17 (0.09-0.31)	107.07/35.7	0.19 (0.14-0.27)
**1-year mortality**								
≥1742.2	4.81/0.0	/	163.14/57.6	0.10 (0.08-0.13)	13.95/2.0	0.03 (0.003-0.11)	96.98/43.4	0.15 (0.11-0.21)
<1742.2	24.29/1.5	0.03 (0.003-0.11)	248.58/56.1	0.07 (0.05-0.09)	36.18/13.3	0.12 (0.06-0.21)	90.13/39.5	0.14 (0.10-0.19)
**ICU admission**								
≥2199.7	0.21/0.0	/	108.93/15.4	1.93 (1.09-3.17)	9.11/0.0	/	78.72/23.5	3.93 (2.53-5.79)
<2199.7	18.82/0.8	0.89 (0.02-4.87)	288.37/59.8	3.37 (2.58-4.31)	45.26/15.1	3.72 (2.10-6.06)	107.29/17.3	1.45 (0.84-2.30)
**Infection**								
≥1930.7	4.49/0.0	/	137.63/8.8	0.91 (0.41-1.72)	12.04/0.5	0.60 (0.02-3.31)	89.62/13.3	0.82 (0.44-1.40)
<1930.7	21.8/0.0	/	290.29/65.7	3.40 (2.63-4.33)	38.03/9.4	1.95 (0.89-3.70)	99.07/7.5	0.44 (0.19-0.87)

[1] Non-users

[2] Cephalosporin monotherapy

[3] Non-cephalosporin users

[4] Cephalosporin combination therapy users

[5] No. at risk/No. of events

[6] Rates/100 participant-day (95%CI)
