# Supplementary figures and images for: The Long-Term Efficacy of Cephalosporin in Elderly Hip Fracture Patients: A Comprehensive Analysis
Source: J Clin Med. 2025 Aug 28;14(17):6086. doi: 10.3390/jcm14176086 (PMC12429005; doi:10.3390/jcm14176086)

A.

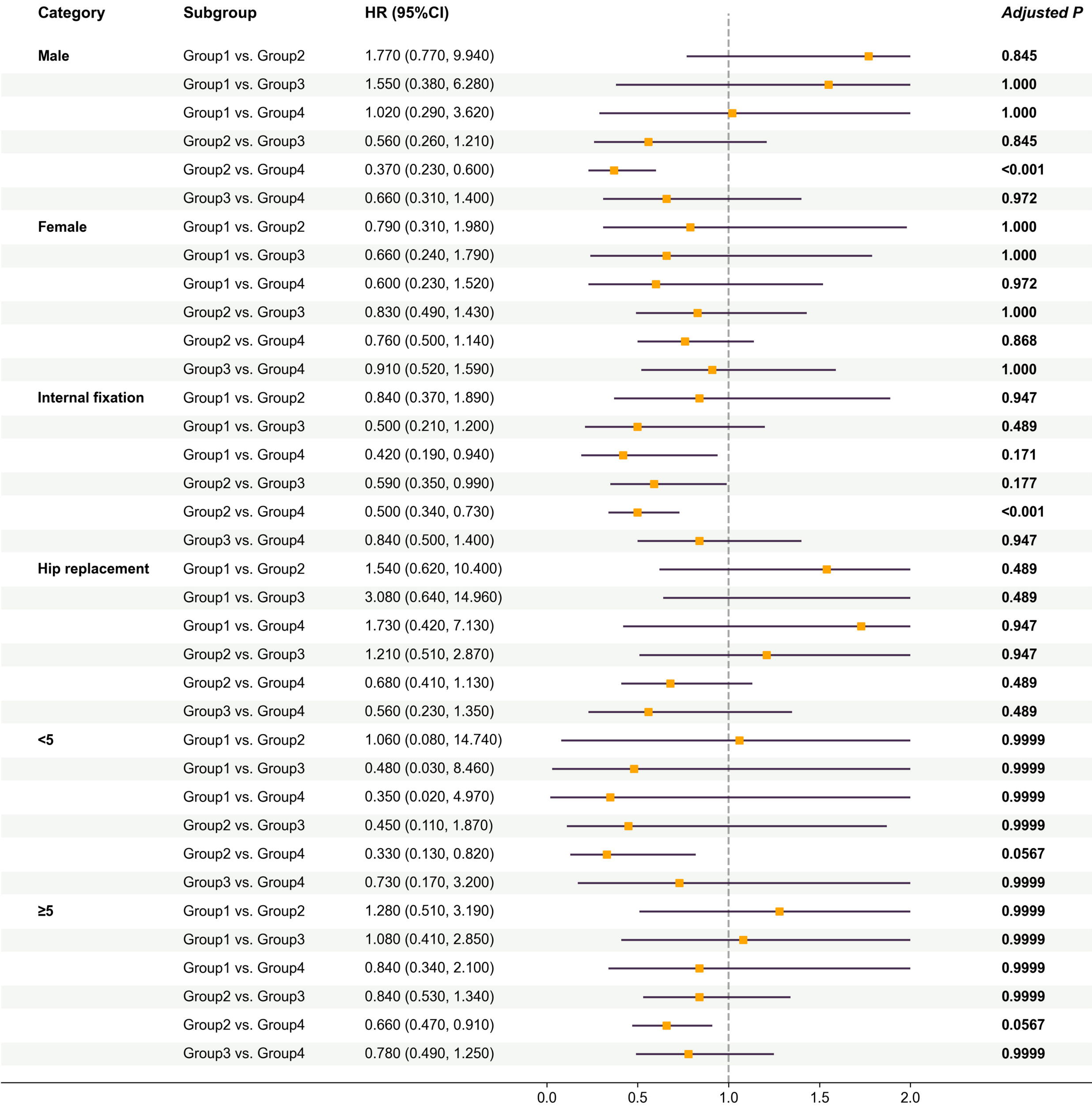

B.

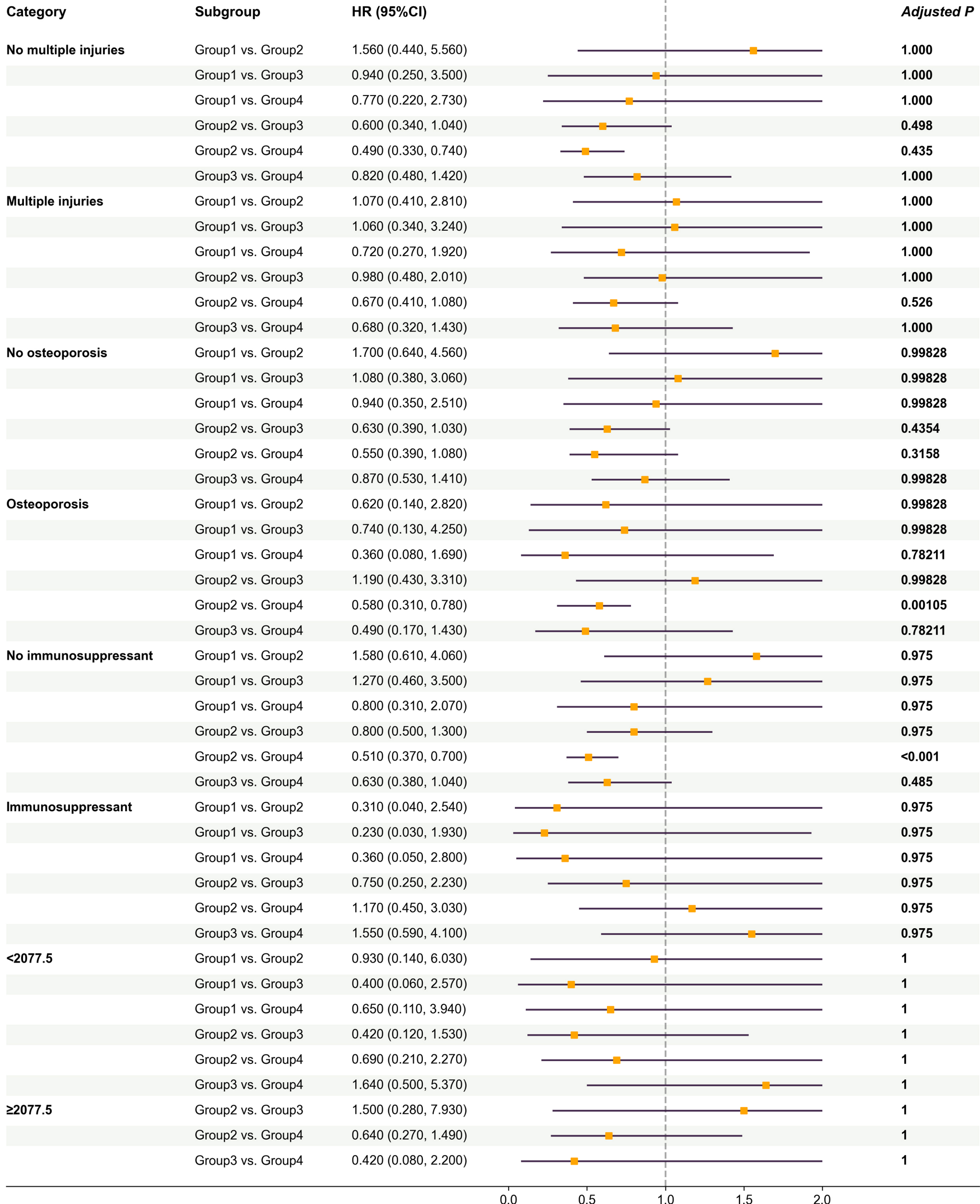

Supplement: Supplementary file 1 [file jcm-14-06086-s001.zip › Supplementary Figure S1.pdf]

A.

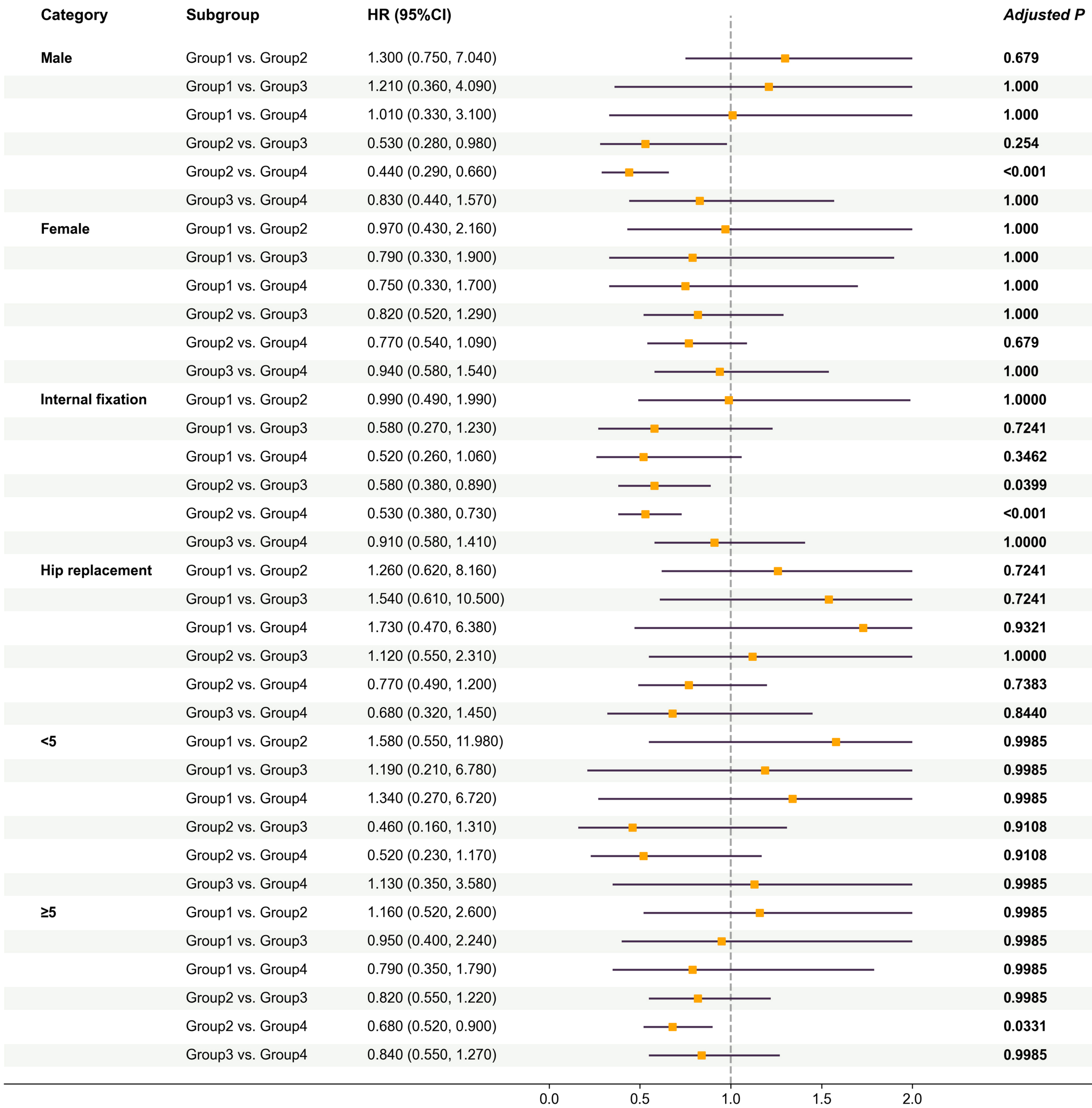

B.

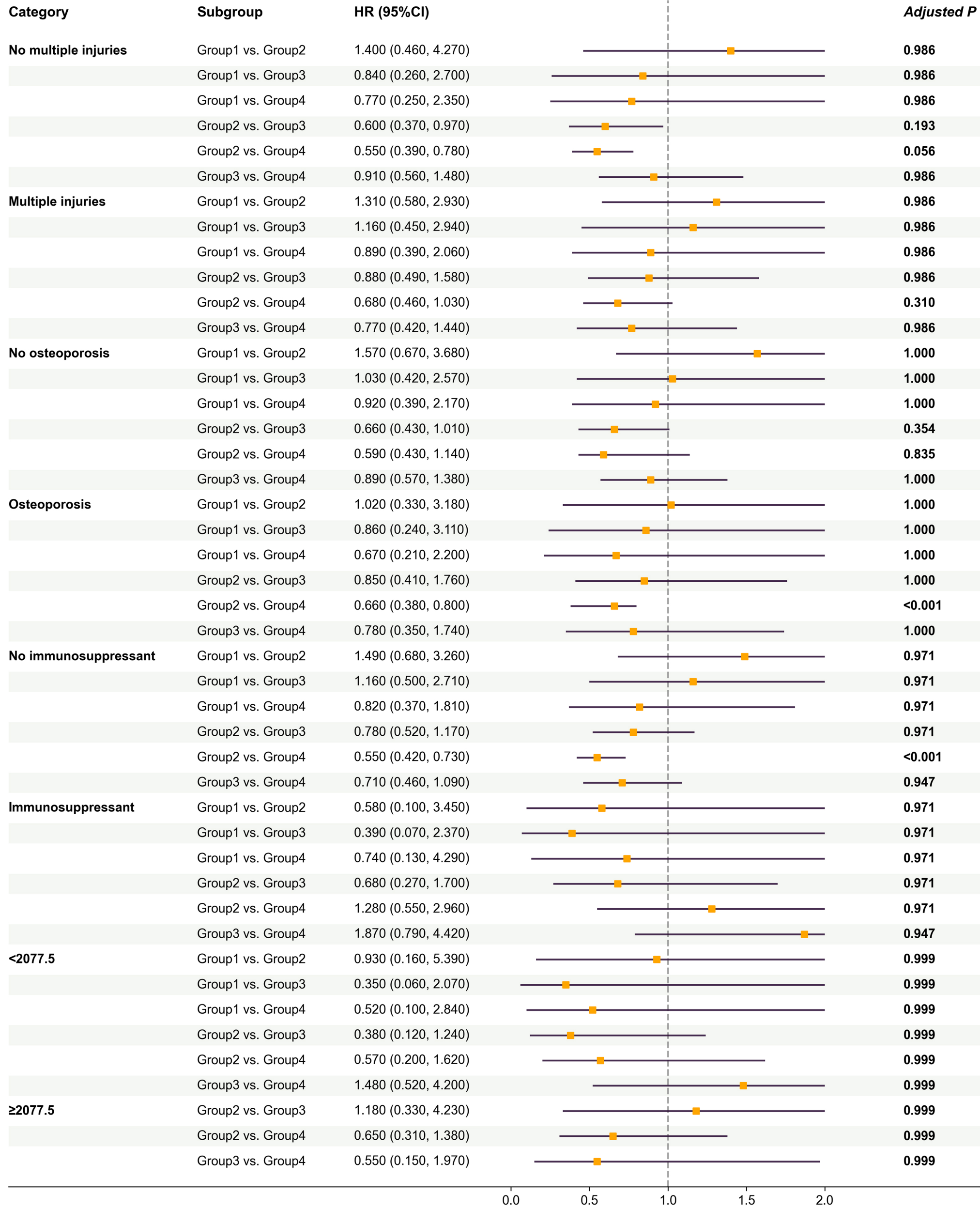

Supplement: Supplementary file 1 [file jcm-14-06086-s001.zip › Supplementary Figure S2.pdf]

A.

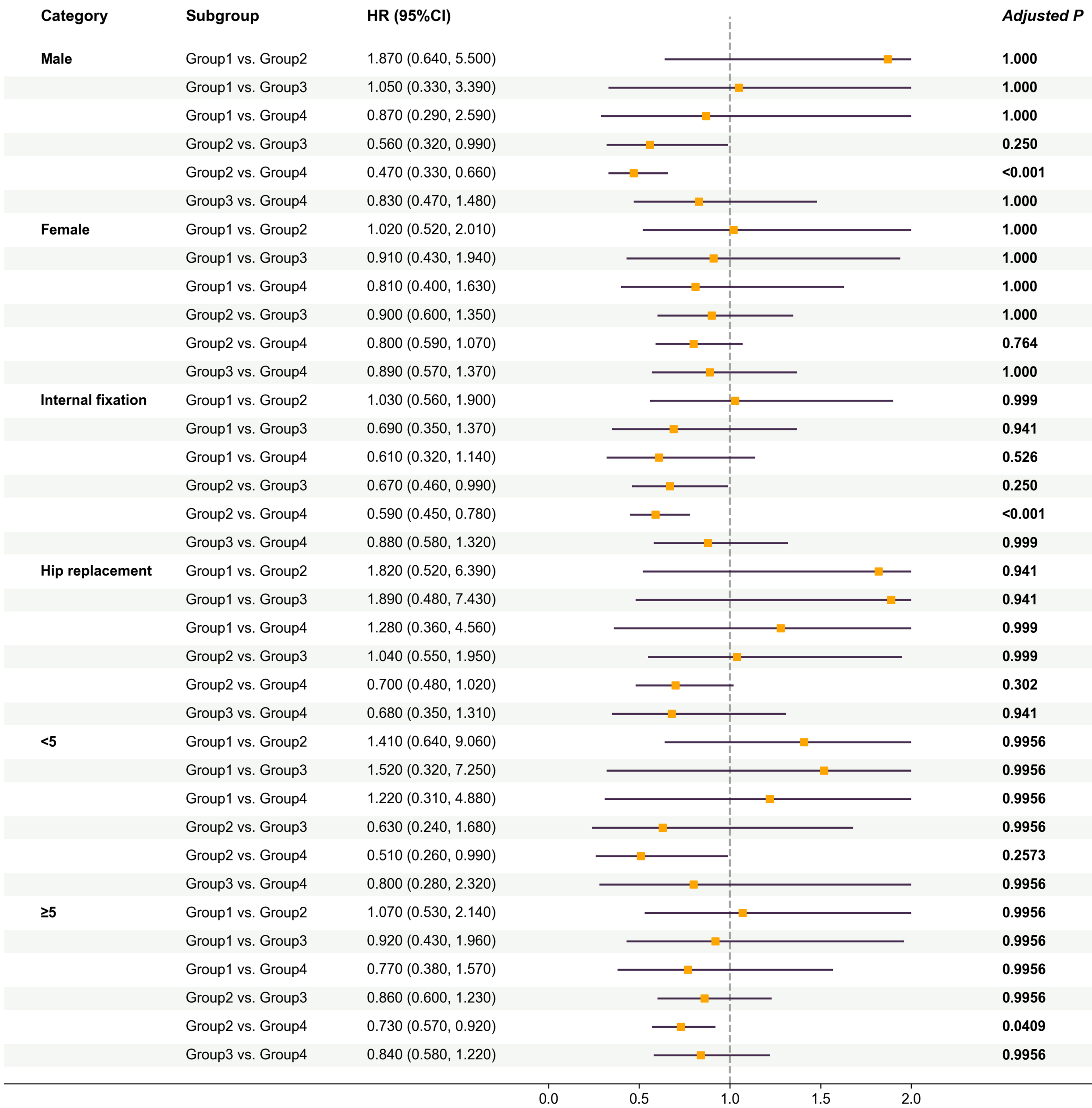

B.

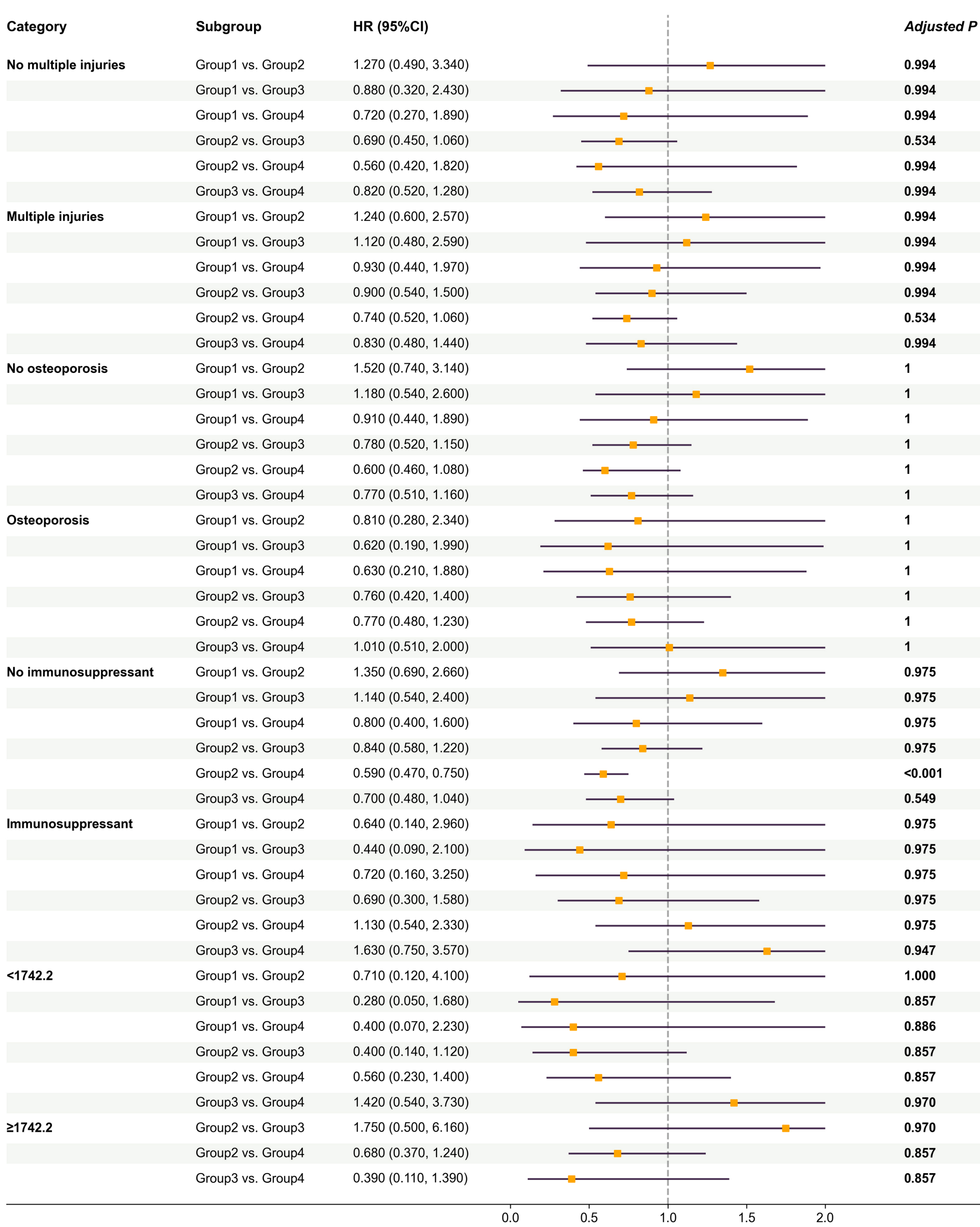

Supplement: Supplementary file 1 [file jcm-14-06086-s001.zip › Supplementary Figure S3.pdf]

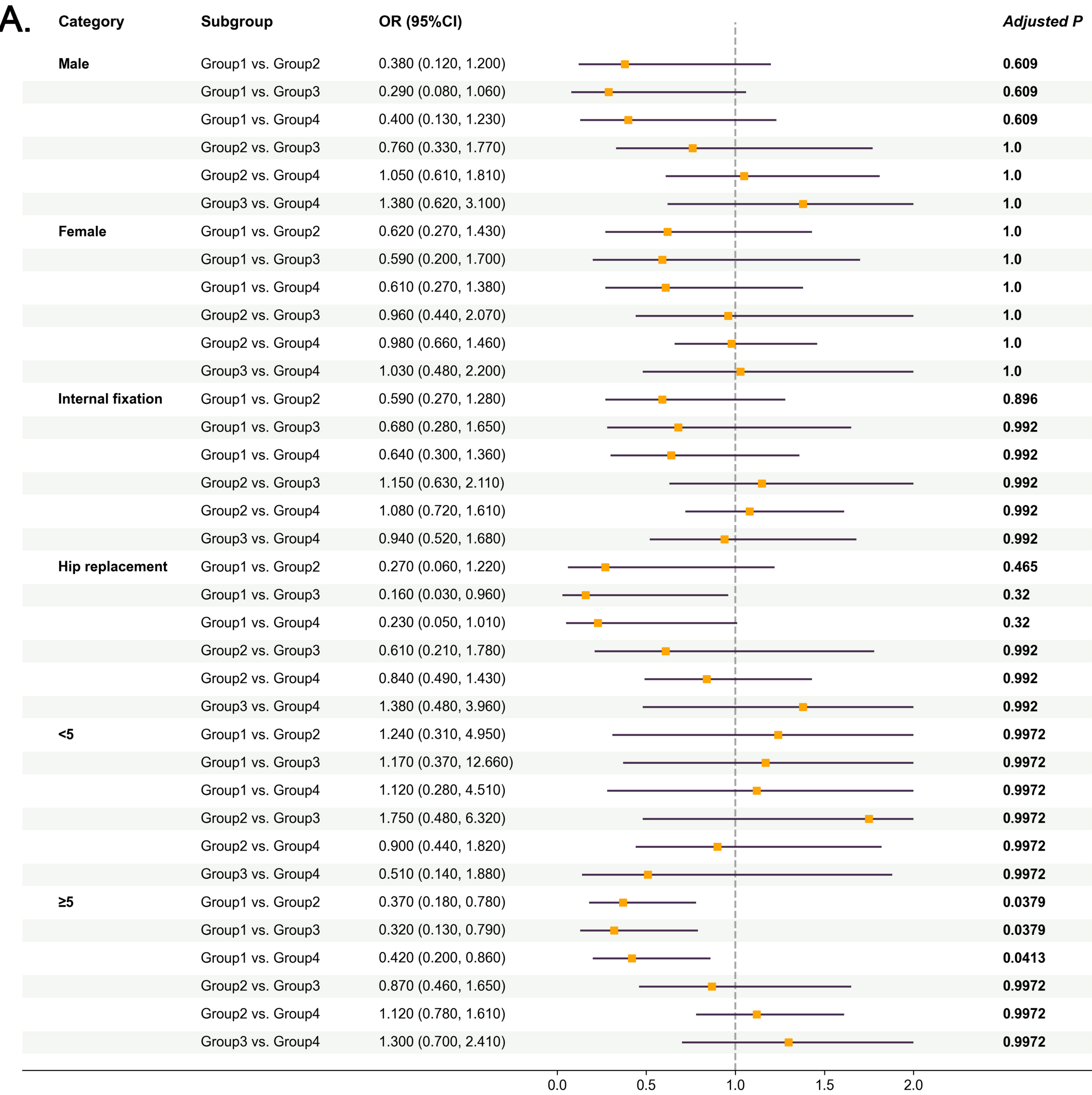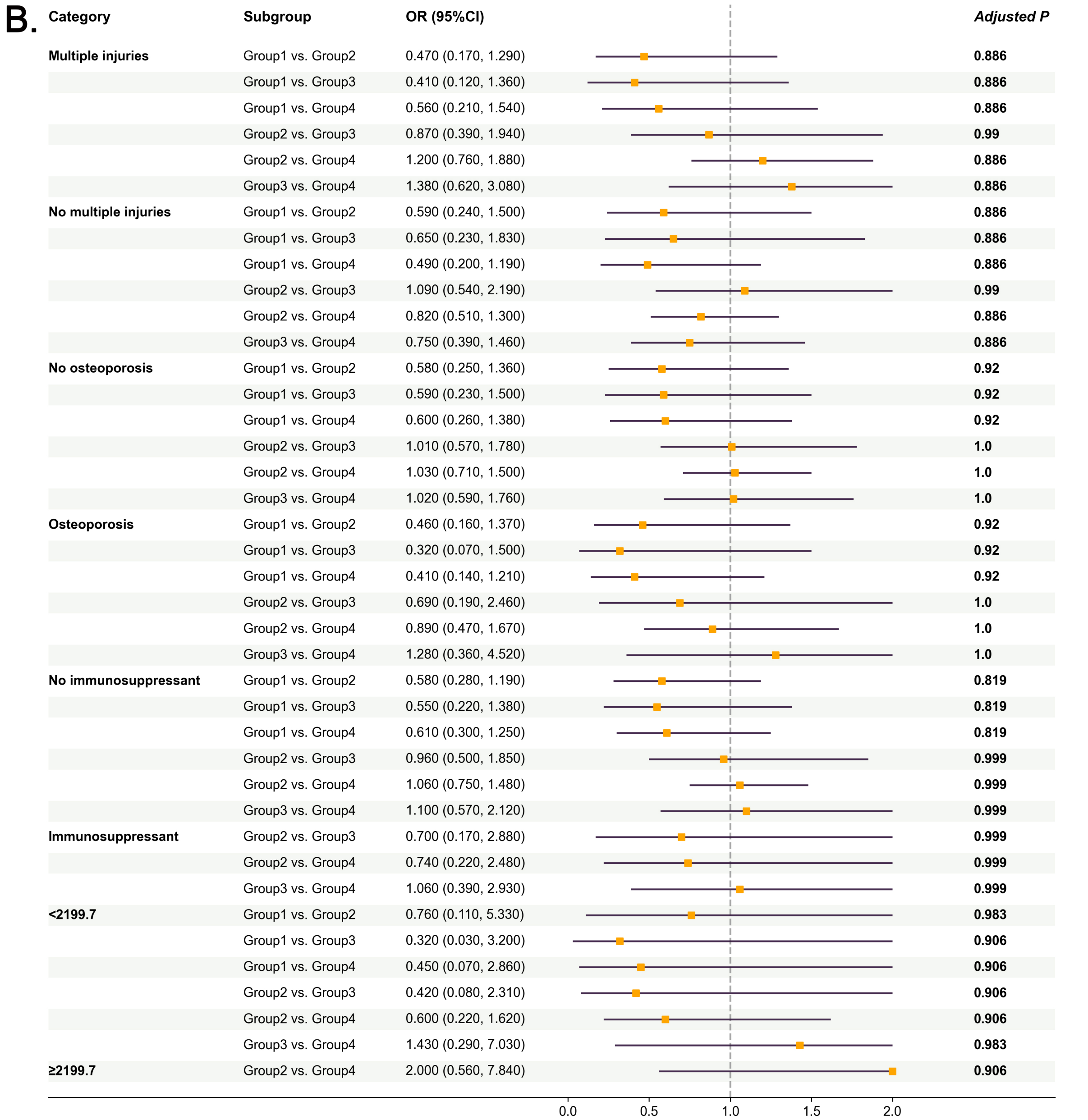

Supplement: Supplementary file 1 [file jcm-14-06086-s001.zip › Supplementary Figure S4.pdf]

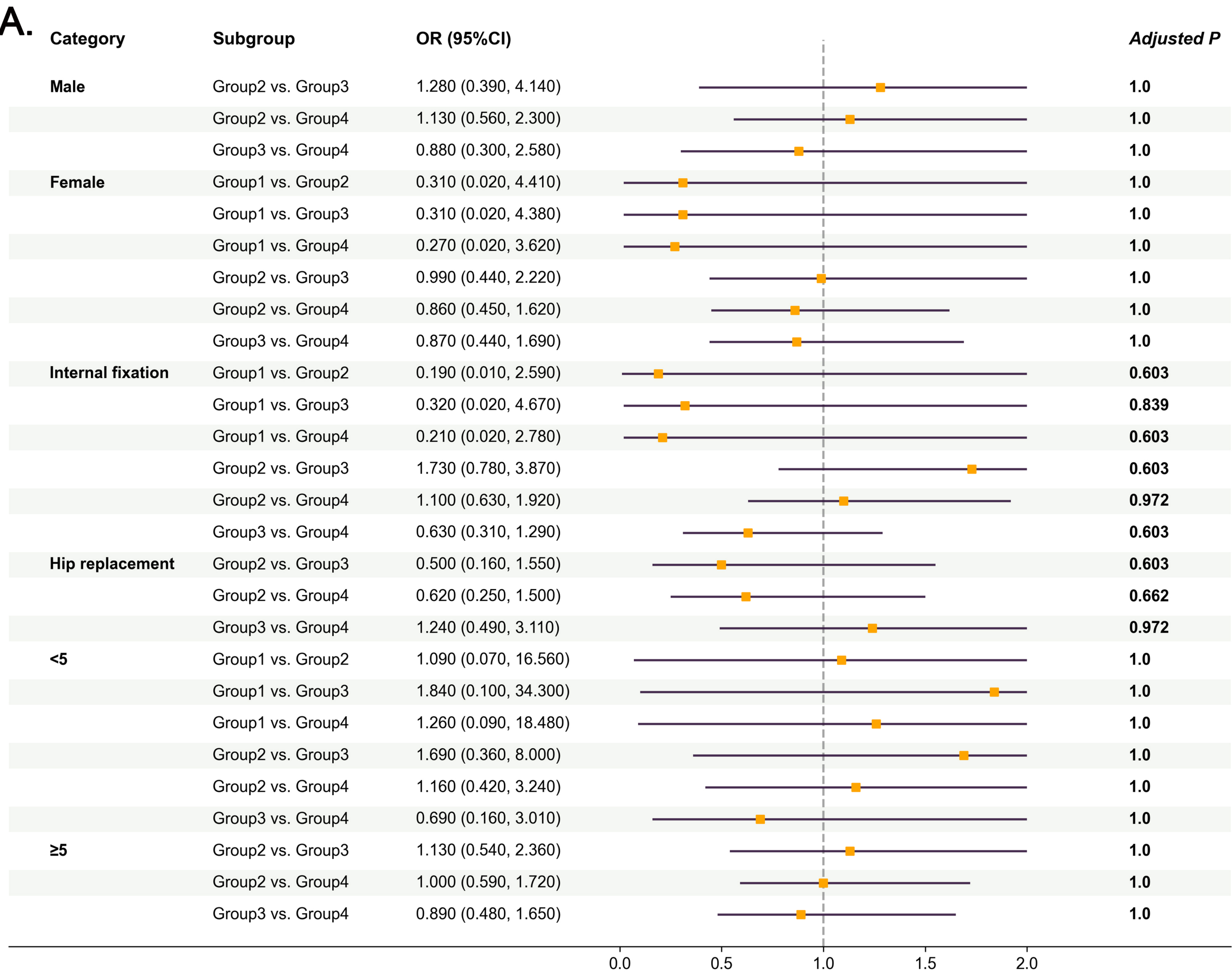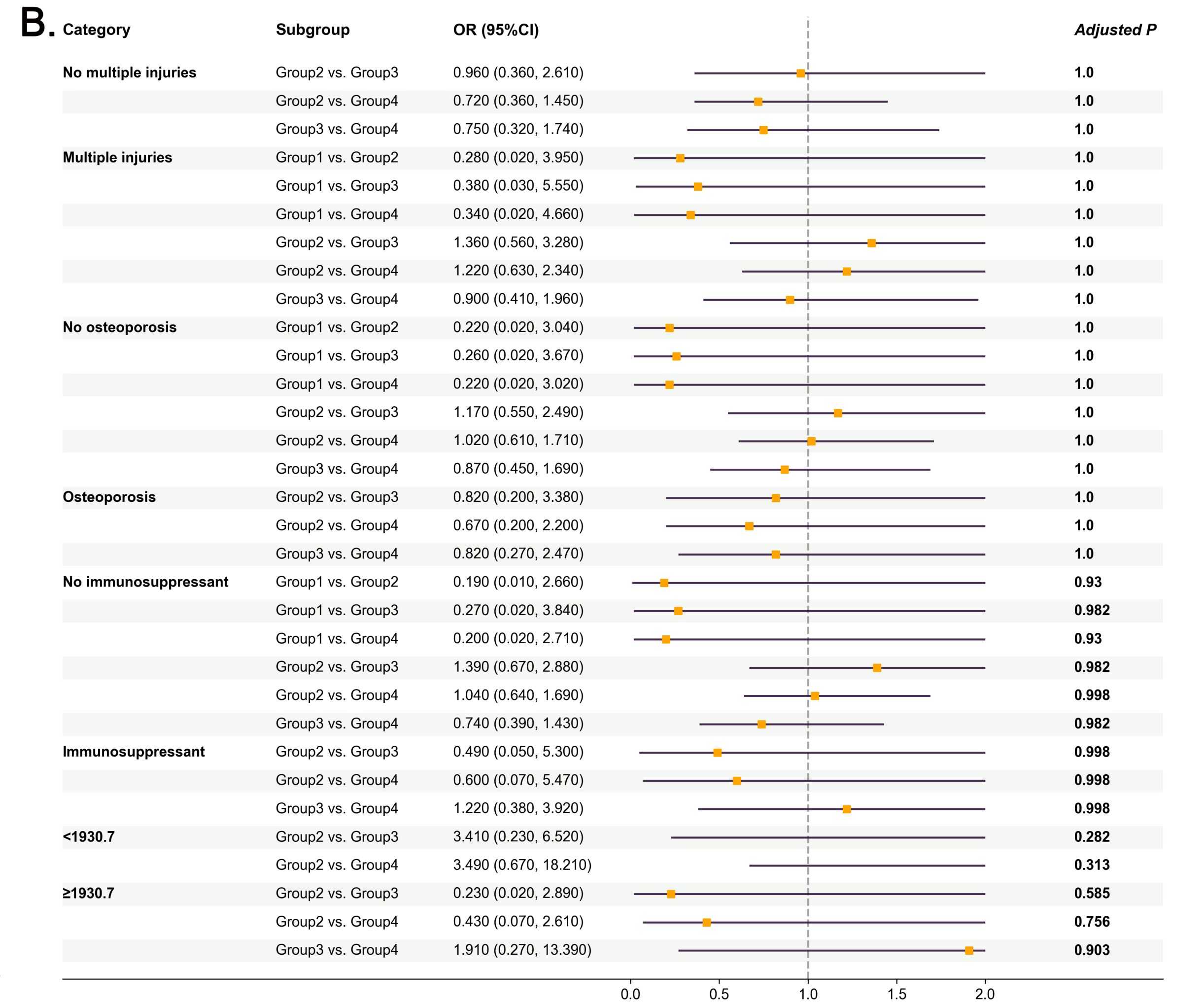

Supplement: Supplementary file 1 [file jcm-14-06086-s001.zip › Supplementary Figure S5.pdf]
